# Tris-Copper Nanozyme as a Novel Laccase Mimic for the Detection and Degradation of Phenolic Compounds

**DOI:** 10.3390/s23198137

**Published:** 2023-09-28

**Authors:** Tong-Qing Chai, Jia-Li Wang, Guo-Ying Chen, Ling-Xiao Chen, Feng-Qing Yang

**Affiliations:** School of Chemistry and Chemical Engineering, Chongqing University, Chongqing 401331, China; 20175531@cqu.edu.cn (T.-Q.C.); 202118021010@cqu.edu.cn (J.-L.W.); 20221801017@stu.cqu.edu.cn (G.-Y.C.); 20175534@cqu.edu.cn (L.-X.C.)

**Keywords:** Tris-copper nanozyme, laccase mimic, colorimetric sensing, degradation, phenolic compound

## Abstract

Phenolic compounds are one of the main organic pollutants in the environment that can seriously affect ecosystems, even at very low concentrations. Due to the resistance of phenolic compounds to microorganisms, conventional biological treatment methods face challenges in effectively addressing this pollution problem. In this study, a novel laccase mimic (Tris-Cu nanozyme) is prepared using a simple and rapid synthesis strategy based on the coordination of copper ions and amino groups in Tris(hydroxymethyl)aminomethane (Tris). It is found that the Tris-Cu nanozyme exhibits good catalytic activity against a variety of phenolic compounds, the *K_m_*, *V_max_* and *K_cat_* are determined to be 0.18 mM, 15.62 μM·min^−1^ and 1.57 × 10^7^ min^−1^ using 2,4-dichlorophenol (2,4-DP) as the substrate, respectively. Then, based on the laccase-like activity of the Tris-Cu nanozyme, a novel colorimetric method for 2,4-DP (the limit of detection (LOD) = 2.4 μM, S/N = 3) detection in the range of 10–400 μM was established, and its accuracy was verified by analyzing tap and lake water samples. In addition, the Tris-Cu nanozyme shows excellent removal abilities for six phenolic compounds in experiments. The removal percentages for 2,4-DP, 2-chlorophenol (2-CP), phenol, resorcinol, 2,6-dimethoxyphenol (2,6-DOP), and bisphenol A (BPA) are 100%, 100%, 100%, 100%, 87%, and 81% at 1 h, respectively. In the simulated effluent, the Tris-Cu nanozyme maintains its efficient catalytic activity towards 2,4-DP, with a degradation percentage of 76.36% at 7 min and a reaction rate constant (*k*_0_) of 0.2304 min^−1^. Therefore, this metal–organic complex shows promise for applications in the monitoring and degrading of environmental pollutants.

## 1. Introduction

With the development of industrialization, the environment has suffered varying degrees of damage, particularly in terms of water resource pollution [1]. Organic pollutants such as phenolic compounds are the main source of water and soil pollution [2]. Phenolic compounds are widely used in many industrial processes, such as in petrochemicals, dyeing, pesticides, and the pharmaceutical and paper industries; the presence of phenolics in industrial effluent makes surface water highly vulnerable to pollution [3,4]. They are a class of pollutants that is highly toxic to humans and animals, even at very low concentrations [5]. In addition, studies have found that elevated concentrations of bisphenol A (BPA) in the urine of adults are associated with obesity and coronary heart disease [6]. Therefore, phenolic compounds such as phenol and resorcinol are included in the list of carcinogens produced by the International Agency for Research on Cancer (IARC) of the World Health Organization. Typically, the concentration of phenolic compounds in wastewater can be as high as several thousand ppm, and the U.S. Environmental Protection Agency (EPA) has set a water purity standard of less than 0.1 ppm [7]. Therefore, it is crucial to detect and remove phenolic contaminants from wastewater before it is discharged. Currently, the conventional detection methods for phenolic compounds are high-performance liquid chromatography, capillary electrophoresis, ion mobility spectrometry, chemiluminescence, continuous flow analysis, flow injection analysis, electrochemistry, and colorimetric sensing [8,9]. These detection methods have excellent accuracy and sensitivity but are expensive and time-consuming and require complex instruments and well-trained technicians; in contrast, colorimetric detection has the advantages of simple operations, low costs, fast speeds, visualization abilities, and on-site detection, and it has great application prospects in environmental pollutant monitoring [10,11].

On the other hand, several techniques have been developed to remove organic pollutants from the wastewater, such as adsorption [12], coagulation [13], extraction [14], biological oxidation [15], photocatalysis [16], electrochemical oxidation [17], and chemical oxidation [8]. Compared to the other sewage treatment methods, chemical catalysis has attracted much attention because of its advantages of simple operations, low costs, and high removal efficiency. Therefore, the development of laccase mimetics, which are inexpensive, simple to prepare, stable, and exhibit highly catalytic activity, has become a research trend. Several metal-based nanomaterials have been found to exhibit laccase-like activity, such as platinum nanoparticles and manganese oxides [18]. Since the active center of natural laccase contains three types of copper ions, many copper-based nanomaterials with laccase-like activity have been reported, such as copper nucleotide/DNA coordination compounds, copper-amino acid/peptide/protein coordination compounds, and other copper-based nanomaterials. For example, Huang et al. synthesized a novel laccase mimicry (AMP-Cu nanozyme) based on the coordination of adenosine monophosphate and copper ions [19]. The study found that the simply prepared AMP-Cu nanozyme has better catalytic activity and robustness than natural laccase and can be recycled via centrifugation. In addition, based on the excellent catalytic performance of the AMP-Cu nanozyme, a colorimetric method was developed for the determination of phenolic compounds in fruit juices using 2,4-dichlorophenol as an example, with a linear range of 0.1–100 μM and a detection limit of 0.033 μM. Furthermore, the AMP-Cu nanozyme was used to remove phenol from the juice; the removal rate was about 65% after a five-hour reaction, which is much higher than that of natural laccase. Wang et al. prepared a novel amorphous imidazole-Cu nanozyme (I-Cu) with high laccase- and catecholase-like activity based on the coordination of Cu^2+^ and N in imidazole via water induction [20]. After 10 h of reaction, the oxidation efficiency of the I-Cu nanozyme for environmental phenolic pollutants (2,4-dichlorophenol) was 91.8%, which is higher than that of laccase (68.8%). However, although many reported laccase-like nanozymes have excellent catalytic activity for phenolic compounds, they still cannot meet the requirements of practical applications, due to factors such as the time-consuming degradation process, low removal efficiency, and lengthy material preparation processes. In addition, the ligands of the various currently reported copper ion complexes are mainly amino acids/peptides/proteins, nucleotides/DNA, and other compounds containing nitrogen heterocycles, such as imidazole, porphyrins, bipyridine, and benzophenone-alanine [18]. However, Tris, a compound with a simple structure and stable properties, has not been reported as a ligand for the preparation of laccase mimics.

In this study, a novel laccase mimic (Tris-Cu nanozyme) based on the coordination of copper ions and amino groups in Tris was prepared for the detection and degradation of phenolic compounds. Tris-Cu nanozyme was prepared rapidly and on a large scale by simply mixing Tris with copper ions, without the need for other organic solvents. Then, the morphology, chemical composition, and chemical valence of the metal elements of the nanozyme were investigated using conventional analytical methods, such as scanning electron microscopy (SEM), transmission electron microscopy (TEM), X-ray diffraction (XRD), Fourier-transform infrared (FT-IR), and X-ray photoelectron spectrometry (XPS). In contrast to natural laccase, the Tris-Cu nanozyme shows excellent catalytic stability within the pH range of 4–11, and NaCl has a significant promoting effect on its catalytic activity. The Tris-Cu nanozyme can catalyze the oxidation of ten phenolic compounds, such as 2,4-dichlorophenol (2,4-DP), phenol, and BPA, and has stronger catalytic activity and substrate affinity than natural laccase. Subsequently, a colorimetric sensing method with a good signal response over a wide detection range was constructed for the detection of phenolic compounds (2,4-DP) in tap and lake water samples. In addition, the Tris-Cu nanozyme shows high efficiency in the removal of six phenolic compounds in phenolic degradation experiments, including 2,4-DP, 2-chlorophenol (2-CP), phenol, resorcinol, 2,6-dimethoxyphenol (2,6-DOP), and BPA. The Tris-Cu nanozyme can quickly remove most of the 2,4-DP from the simulated sewage.

## 2. Materials and Methods

The materials and reagents, instrumentation, selective studies, 4-aminoantipyrine spectrophotometry method for the detection of phenolic compounds, preparation of simulated sewage, method for calculating the relative activity, and limit of detection (LOD) are provided in the Supplementary Materials.

### 2.1. Preparation of the Tris-Cu Nanozyme

The Tris-Cu nanozyme was prepared through a simple synthesis strategy. Frist, 1 mL of CuSO_4_·5H_2_O (0.2 M) aqueous solution and 1 mL of Tris (0.2 M) aqueous solution were mixed in centrifuge tubes, and the blue pellets were obtained after centrifugation at 4000 rpm for 2 min. Then, the precipitation was washed three times with pure water and centrifuged for 2 min each. Finally, 1 mL of the NaAc buffer (10 mM, pH 6.0) was added to resuspend the precipitate.

### 2.2. Evaluation the Catalytic Activity of the Tris-Cu Nanozyme and Laccase

In brief, 20 μL of the Tris-Cu nanozyme (11 mg/mL), 100 μL of the 4-AP (1.0 mM), and 100 μL of the 2,4-DP (1.0 mM) solutions were added to 280 μL of NaAc buffer (10 mM, pH 6.0), and they were then reacted at 70 °C for 10 min. Then, the reaction mixture was centrifuged at 4000 rpm for 2 min and the absorbance at 510 nm of the supernatant was recorded by a UV–Vis spectrometer. In addition, the catalytic activity of laccase (11 mg/mL, 100 μL) was investigated under the same conditions.

To study the reaction kinetics, Tris-Cu (20 μL) was added into different concentrations of 2,4-DP (0.005–0.6 mM) in the presence of 4-AP (0.2 mM), and the mixture was reacted under the optimal conditions (pH 6.0, 70 °C, 10 min). The apparent initial reaction velocity was measured. The kinetic parameters (*K_m_* and *V_max_*) were calculated via the Michaelis–Menten equation [19,20]. Furthermore, the *K_cat_* of the Tris-Cu nanozyme was measured according to the method reported by Jiang et al. [21]:

(1) $$K_{cat}=V_{max}/[E]$$

(2) $$[E]=\rho_{e}/\left(m_{s}\times N_{A}\right)$$

(3) $$m_{s}=\rho_{s}\times \pi d^{3}\times 10^{-21}/6$$

where *K_cat_* is the rate constant defining the maximum number of substrate molecules converted to product per unit of time, [*E*] is the molar concentration of the nanozyme, *ρ_e_* is the mass concentration of the nanozyme (g/L), *m_s_* is the mass of a single nanozyme particle (g), *N_A_* is Avogadro’s constant (6.02 × 10^23^ mol^−1^), *ρ_s_* is the density of the nanozyme (g/cm^3^), and *d* is the average diameter of a nanozyme particle (nm) obtained through the statistical analysis of more than 100 nanoparticles in a SEM image.

To measure the density of the nanozyme, 100 mg of the Tris-Cu nanozyme was compressed as a tablet 13.0 mm in diameter and 0.30 mm in height under 14 MPa for 2 min. Subsequently, the volume of the tablet was calculated to be 39.80 mm^3^ and the mass of the tablet was again measured as 98.0 mg. Therefore, the density of the Tris-Cu nanozymes was calculated to be 2.46 g/cm^3^.

### 2.3. Robustness of the Tris-Cu Nanozyme

To investigate the effect of salt on the stability of the Tris-Cu nanozyme and laccase, various concentrations of NaCl (0, 150, 300, and 500 mM) were added and incubated for 12 h. Afterwards, the changes in the catalytic activity of the Tris-Cu nanozyme and laccase were investigated under the optimal conditions. Furthermore, to study the effect of ethanol on the activity of the Tris-Cu nanozyme and laccase, various volumes of ethanol (volume fraction φ: 0%, 10%, 20%, 30%, and 40%) were added to the reaction solutions. Then, the changes in the activity of Tris-Cu nanozyme and laccase were studied under the optimal conditions. For the study of long-term storage stability, the Tris-Cu nanozyme and laccase in the NaAc buffer (10 mM, pH 6.0) were stored at 2 °C for 8 days, and the changes in their activity were studied under optimal conditions.

### 2.4. Catalytic Mechanism of the Tris-Cu Nanozyme

To study the role of reactive oxygen species (ROS) on the catalytic process of the Tris-Cu nanozyme, various concentrations (0, 5, 25, and 50 mM) of the superoxide anion radical scavenger 1,4-benzoquinone and the hydroxyl radical scavenger isopropanol (IPA) were added to the reaction mixture. Furthermore, O_2_ and N_2_ were introduced into the reaction system to investigate the catalytic mechanism of the Tris-Cu nanozyme. The activity of the Tris-Cu nanozyme was observed under the optimal conditions.

### 2.5. Detection of Phenolic Compounds in Water Samples

To facilitate quantitative detection, phenolic compounds were preliminarily identified using colorimetry analysis. Different concentrations of phenolic compounds (0.2, 0.4, and 0.8 mM) were oxidized in the presence of 4-AP (0.2 mM) by the Tris-Cu nanozyme under the optimal conditions (pH 6.0, 70 °C, 10 min) and their color changes were recorded. Moreover, to investigate the selectivity of the Tris-Cu nanozyme, Tris-Cu was used to catalyze analogues of phenols. In brief, in the presence of 4-AP (0.2 mM), the Tris-Cu nanozyme oxidizes phenol, methylbenzene, methylbenzene, benzyl alcohol, 4-nitrobenzaldehyde, 4-methoxybenzoic acid, and 4-aminobenzoic acid (0.2 mM) under optimal conditions (pH 6.0, 70 °C, 10 min). Then, the reaction mixture was centrifuged at 4000 rpm for 2 min and the absorbance of the supernatant at 510 nm was recorded using a UV–Vis spectrometer. The method used for the colorimetric detection of phenolic compounds (2,4-DP, for example) was constructed based on using the Tris-Cu nanozyme as a laccase mimic. In brief, various concentrations of 2,4-DP (10–400 μM) were oxidized in the presence of 4-AP (0.2 mM) by the Tris-Cu nanozyme and the mixture solution was reacted under the optimal conditions. Then, the relationship between the 2,4-DP concentration and the corresponding absorption intensity at 510 nm was investigated. To study the specificity and selectivity of the colorimetric sensors, the laccase-like activity of the Tris-Cu nanozyme was examined in the presence of different interfering substances (1 mg/mL), such as Na_2_SO_4_, NaNO_2_, CuNO_3_, ZnSO_4_, MgSO_4_, CoNO_3_, Na_2_CO_3_, NaHCO_3_, KIO_3_, CaCO_3_, glucose, fructose, and BSA. Furthermore, the developed colorimetric method was used to detect 2,4-DP in tap and lake water samples. In brief, different concentrations of 2,4-DP (0, 10, 100, and 400 μM) were added to both tap and lake water, followed by addition of the Tris-Cu nanozyme (20 μL), 4-AP (0.2 mM), and the NaAc buffer (10 mM, 6.0) into the solution. The total volume of the mixture was maintained at 500 μL. Finally, the content of 2,4-DP in the sample was detected using the developed method.

### 2.6. Degradation of Phenolic Compounds

To study the efficiency of the removal of phenolic compounds by the Tris-Cu nanozyme, the solutions of different phenolic compounds (2,4-DP, 2-CP, phenol, resorcinol, 2,6-DOP, BPA; 0.5 mM, 100 μL), 4-AP (1.0 mM, 100 μL), and Tris-Cu nanozyme (11 mg/mL, 20 μL) were added into the NaAc buffer (10 mM, pH 6.0, 280 μL). Then, the mixtures were reacted at 70 °C for 0–60 min and centrifuged at 4000 rpm for 2 min, and the absorbances at the maximum absorption wavelength (510 nm) of the supernatant were recorded by a UV–Vis spectrometer. Finally, the content of the residual phenolic compounds in the supernatant was detected using the 4-aminoantipyrine spectrophotometry method:

(4) $$\mathrm{Removal}\;\mathrm{rate}\;(\%)=(c_{0}-c_{r})/c_{0}\times 100\%$$

where *c_r_* and *c*_0_ are the residual and initial concentrations of phenolic compounds, respectively. Afterwards, to investigate the potential of this method in practical applications, the removal of 2,4-DP (0.1 mM) from simulated sewage by the Tris-Cu nanozyme was investigated in the presence of 4-AP (0.2 mM). The reaction kinetics of 2,4-DP were observed via a first-order kinetic model, expressed as follows:

(5) $$ln\frac{C_{0}}{C_{t}}=k_{1}t$$

where *C*_0_ is the initial concentration of 2,4-DP in the simulated sewage, *C_t_* is the concentration of 2,4-DP at time *t* (min), and *k*_1_ is the observation of the first-order reaction rate constant (min^−1^).

## 3. Results and Discussion

### 3.1. Characterization of the Tris-Cu Nanomaterial

**Figure 1:**
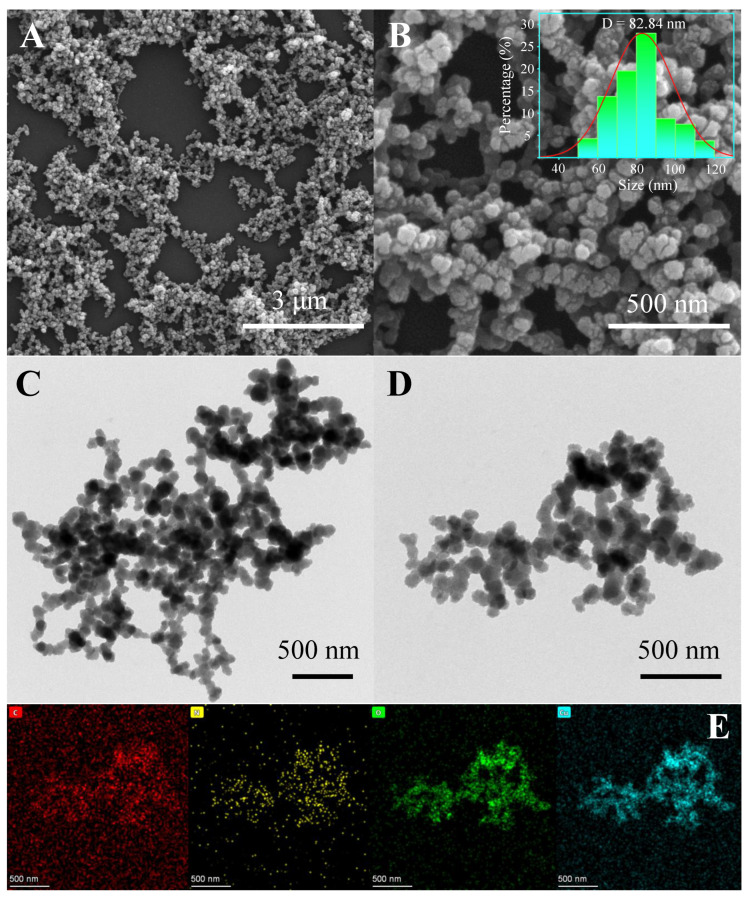
SEM images (**A**,**B**) and the particle size distribution (inset), TEM images (**C**,**D**), and EDS elemental mapping images (**E**) of the Tris-Cu nanozyme.

The morphology of the prepared Tris-Cu nanomaterial was observed via SEM and TEM. As shown in Figure 1A,B, Tris-Cu is constructed of cross-linked nanoparticles with a particle size mainly distributed around 82.84 nm. Figure 1C,D shows that there are many voids between the interlinked Tris-Cu nanoparticles, providing abundant binding sites for the substrate and reducing mass transfer resistance during catalysis. The elemental mapping images (Figure 1E) clearly show that the four elements of carbon, nitrogen, oxygen, and copper are uniformly distributed in the Tris-Cu nanoparticles, which indicates its successful synthesis.

**Figure 2:**
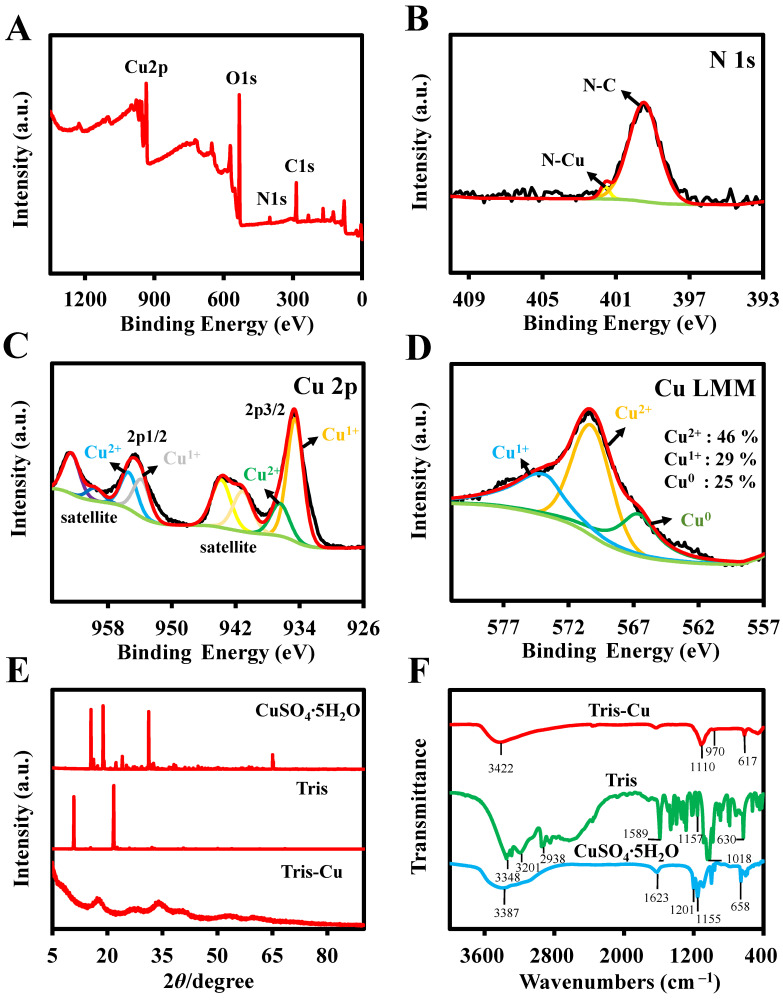
XPS analysis of the AP-Cu nanozyme: element survey (**A**); N 1s (**B**); Cu 2p (**C**); Cu LMM (**D**). (**E**) XRD patterns of CuSO_4_·5H_2_O, Tris, and Tris-Cu. (**F**) FT-IR spectra of Tris-Cu (red line), Tris (green line), and CuSO_4_·5H_2_O (blue line).

XPS analysis was performed to further investigate the elemental composition and atomic valence state of the Tris-Cu nanoparticles (Figure 2A–D and Figure S1). The XPS survey spectra (Figure 2A) show the signals of Cu 2p, O 1s, N 1s, and C 1s, which are consistent with the elemental mapping images. The contents of copper, oxygen, nitrogen, and carbon are 14.98%, 45.17%, 4.54%, and 35.31%, respectively (Table S1). As shown in Figure S1A, the high-resolution spectra of C 1s attributed to the peaks of the C-C and C-O bonds are at 284.80 eV and 288.38 eV, respectively. Only one peak (531.38 eV) attributed to the O-C bond is in the O 1s resolution spectrum (Figure S1B). The high-resolution spectrum of N 1s (Figure 2B) shows two peaks at 399.48 eV and 401.58 eV, corresponding to the N-C and N-Cu bonds, respectively, which shows that the Tris-Cu nanoparticles are synthesized through coordination between the nitrogen atoms of Tris and coppers. In the fine XPS spectrum of Cu 2p (Figure 2C), the peaks at 937.28 eV (Cu 2p3/2) and 955.38 eV (Cu 2p1/2) are associated with Cu^2+^, and the peaks at 934.18 eV (Cu 2p3/2) and 953.98 eV (Cu 2p1/2) indicate the presence of Cu^1+^. To further investigate the valence state of the Tris-Cu nanoparticles, an Auger spectrum of Cu LMM was produced (Figure 2D). The three main peaks at 566.64 eV, 570.24 eV, and 573.83 eV are associated with Cu^0^, Cu^2+^, and Cu^+^, respectively. The contents of Cu^0^, Cu^2+^, and Cu^+^ were determined to be 25%, 46%, and 29% via the ratio of the integral corresponding peak area, respectively. The presence of Cu^0^ and Cu^+^ in the Tris-Cu nanomaterials may be due to the reduction of Cu^2+^ during synthesis [20]. Furthermore, as shown in Figure 2E, the crystal structure of the Tris-Cu nanoparticles is further studied through XRD characterization. Unlike Tris and CuSO_4_·5H_2_O, the diffraction peaks of Tris-Cu are wide and rounded, which demonstrates that the Tris-Cu nanozyme may be amorphous. To further investigate the chemical bonds in the Tris-Cu nanozyme, FT-IR analysis was performed (Figure 2F and Table S2). The peaks at 3201 cm^−1^, 2938 cm^−1^, and 1589 cm^−1^ correspond to the stretching vibrations of the aminos in Tris, and their disappearance in the spectrum of the Tris-Cu nanoparticles indicates that Cu ions are coordinated with the nitrogen atoms of amino groups. The stretching vibrations at 1110 cm^−1^ and 1157 cm^−1^ indicate the presence of C-N in Tris and Tris-Cu. The absorptions at 970 cm^−1^ and 1018 cm^−1^ are the stretching vibrations of C-O and the absorptions at 617 cm^−1^ and 630 cm^−1^ are the out-of-plane bending vibrations of C-O. In addition, the characteristic absorption peaks of SO_4_^2+^ are not exhibited in Tris-Cu. These results prove that the Tris-Cu nanozyme is a new material synthesized through the coordination of Tris with copper ions.

### 3.2. Evaluation of the Activity of the Tris-Cu Nanozyme and Laccase

**Figure 3:**
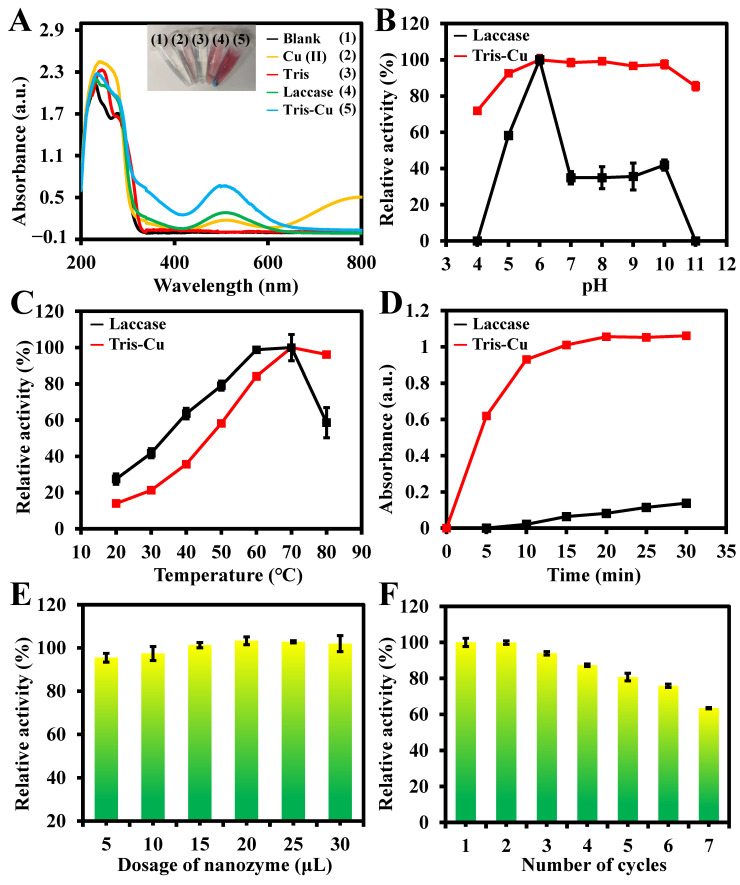
(**A**) UV–Vis absorption spectra of a mixture of the NaAc buffer (10 mM), CuSO_4_ (40 mM), Tris (40 mM), laccase (10 mg/mL, 200 U/g), and the Tris-Cu nanozyme (100 μL) added to 2,4-DP (0.2 mM) and 4-AP (0.2 mM). The relative activity of the Tris-Cu nanozyme and laccase varies according to the pH (**B**), temperature (**C**), time (**D**), and dosage of the Tris-Cu (**E**). Reusability of the Tris-Cu nanozyme (**F**) (conditions: Tris-Cu nanozyme 20 μL, 2,4-DP 0.2 mM, 4-AP 0.2 mM, pH 6.0, 70 °C, 10 min).

The laccase-like activity of the Tris-Cu nanozyme was evaluated based on the oxidation of 2,4-DP in the presence of 4-AP. Figure 3A shows that Tris-Cu has outstanding laccase-like activity compared to copper ions and natural laccase. To optimize the preparation conditions of the Tris-Cu nanozyme, the feeding ratio of Tris to copper ions and the concentration of copper ions during synthesis were investigated (Figure S2). We found that the laccase-like activity of the Tris-Cu nanozyme gradually increased with the increase in the Tris/Cu ratio until reaching 1:1. Moreover, with the increase in the copper ion concentration, the yield of the Tris-Cu nanozyme is increased. Therefore, to simply and quickly prepare a nanozyme with high catalytic activity, the Tris-Cu nanomaterial was prepared with the feeding ratio of 1 and copper ions of 100 mM. In addition, the effects of different buffer systems on the catalytic activity of Tris-Cu were compared (Figure S3), and the results show that the Tris-Cu nanozyme has the best catalytic activity in the NaAc buffer, which was selected for the subsequent experiments. According to the literature [22,23,24], pKa is 7.51 for HEPES, 7.21 for PBS, 7.0 for H_2_O, 6.15 for MES, and 4.76 for NaAc. At pH = 7, HEPES and PBS are positively charged, while MES and NaAc are negatively charged. The difference in catalytic activity may be because negatively charged NaAc is more conducive to the binding of the Tris-Cu nanozyme to phenolic compounds in this buffer, thereby improving the catalytic activity. As illustrated in Figure 3B–E, like natural laccase, the catalytic activity of the Tris-Cu nanozyme is affected by the pH, temperature, reaction time, and dosage of the nanozyme. The natural enzyme is completely inactive at pH values of 4 and 11, but the relative activity of the Tris-Cu nanozyme remains above 70%, indicating that the Tris-Cu nanozyme has better acid and alkali resistance. As shown in Figure S4, in order to further investigate the alkali resistance of the Tris-Cu nanozyme, the Zeta potential of the Tris-Cu nanozyme in the NaAc buffer at different pH values was investigated. The results show that the Zeta potential of the Tris-Cu nanozyme is stable between 18.18 mV and 20.64 mV in the pH range of 6–10. It is worth mentioning that even the Zeta potential of the Tris-Cu nanozyme changes significantly in an acidic or strongly alkaline environment, while the catalytic activity of Tris-Cu does not decrease significantly. This may be due to the harsh environment that disrupts the microstructure of the Tris-Cu nanozyme but has little effect on the structure of the catalytic center. This further demonstrates the stability of the catalytic activity of the Tris-Cu nanozyme. Furthermore, the catalytic activity of the Tris-Cu nanozyme and natural laccase shows the same trend with the increase in the temperature (20–80 °C): that is, it first increased with the increase in temperature and then decreased when the temperature reached 70 °C. However, the Tris-Cu nanozyme can retain above 96% of its maximum catalytic activity when the temperature reaches 80 °C. In addition, as shown in Figure 3D,E, the activity of Tris-Cu increases with increasing reaction time and higher dosage of nanozyme. Therefore, subsequent experiments were performed under optimal conditions: pH 6.0, 70 °C, 10 min, 20 μL of nanozyme (11 mg/mL), total volume of 500 μL.

To further study the laccase-like activity of the Tris-Cu nanozyme, the kinetic parameters (*K_m_* and *V_max_*) are calculated by catalyzing different concentrations of 2,4-DP in the presence of the Tris-Cu as the catalyst (Figure S5). The *K_m_* and *V_max_* of the Tris-Cu nanozyme are calculated to be 0.18 mM and 15.62 μM·min^−1^, respectively. The *K_m_* value is negatively correlated with the strength of the affinity between the enzyme and substrate, and a smaller *K_m_* represents a stronger affinity [25]. The results show that the affinity and catalytic activity of the Tris-Cu nanozyme in relation to the substrate are 2.3 times and 2.4 times higher than that of laccase, respectively (Table 1). Compared to other nanozymes, the catalytic activity and substrate affinity of the Tris-Cu nanozyme also have obvious advantages. In addition, the *K_cat_* (1.57 × 10^7^ min^−1^) and *K_cat_*/*K_m_* (8.72 × 10^7^ mM^−1^ min^−1^) of the Tris-Cu nanozyme are much higher than those of laccase and other nanozymes. The *K_cat_* and *K_cat_*/*K_m_* of the Tris-Cu nanozyme are 9.1 times and 22.7 times higher than that of copper fumarate (Cu FMA). This indicates that Tris-Cu nanoparticles have a higher turnover number and catalytic efficiency, and they have a higher number of active sites than laccase and other laccase mimics. This may be related to Tris-Cu’s cross-linked nanostructure; there are many voids between the interconnected Tris-Cu nanoparticles, which provide abundant binding sites for the substrate and reduce the resistance to mass transfer during catalysis.

### 3.3. Catalytic Stability and Reusability of the Tris-Cu Nanozyme

Reusability is one of the obvious advantages of laccase mimicries compared to natural laccase. Tris-Cu nanozyme can be recycled via centrifugation, which reduces the cost of practical applications. As shown in Figure 3F, reusability experiments show that the catalytic activity of the Tris-Cu nanozyme remains above 80% after repeated use (five times), while the activity remains above 63% after seven repeated uses. Therefore, the Tris-Cu nanozyme can be reused at least five times with good activity.

**Figure 4:**
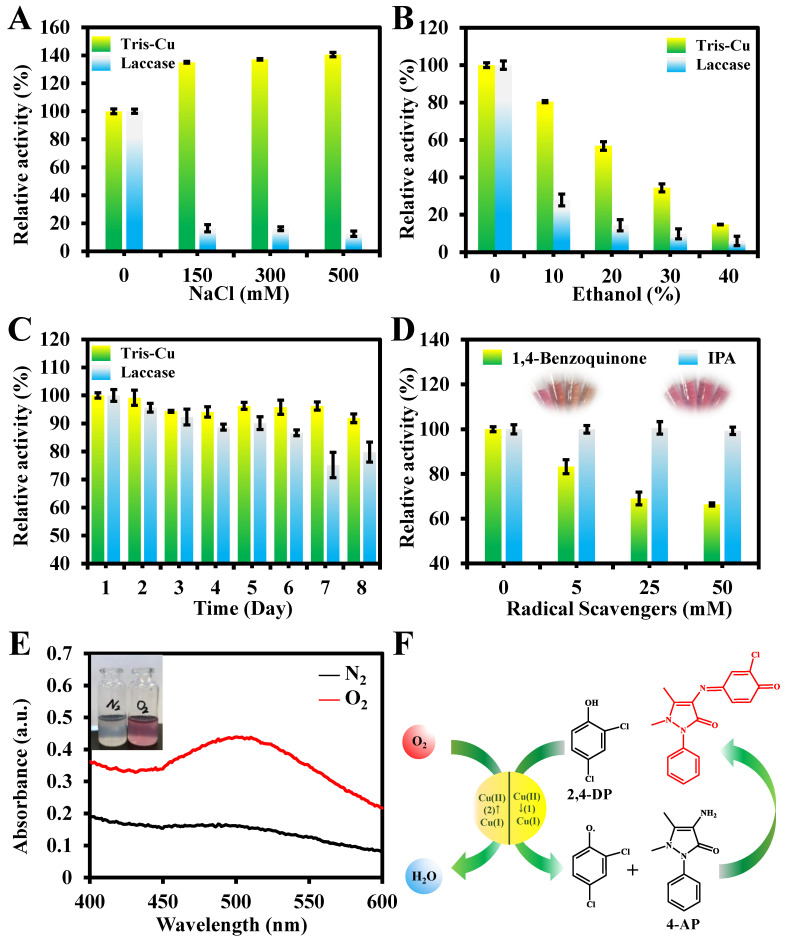
Effects of different concentrations of NaCl (**A**), ethanol (**B**), and storage times (**C**) on the catalytic activity of the Tris-Cu nanozyme and laccase. Effect of various concentrations of 1,4-benzoquinone and IPA on the catalytic performance of the Tris-Cu nanozyme (**D**). UV–Vis absorption spectra of the Tris-Cu-nanozyme-catalyzed laccase reaction under oxygen and nitrogen (100 mL/min) (**E**). Possible catalytic mechanism of the Tris-Cu nanozyme (**F**).

To study the practical applications of the Tris-Cu nanozyme, the effects of Cl ions and ethanol content on the catalytic activity of the Tris-Cu nanozyme and natural laccase are investigated, respectively. As shown in Figure 4A, Cl ions can promote the oxidation of the substrate by the Tris-Cu nanozyme, which is consistent with the findings reported in the literature [26,32,33,34]. The reason for this phenomenon is that Cl ions, as inhibitors of laccase, can bind to the type 2/3 (T2/T3) trinuclear copper site of laccase, resulting in the inactivation of laccase [35]. However, a high concentration of sodium chloride will reduce the solubility of the substrate in the system and promote the binding of the substrate to the nanozyme, thus greatly increasing the catalytic activity [19]. In addition, it is found that the catalytic activity of both the Tris-Cu nanozyme and laccase show a significant decrease with the increase in the ethanol concentration in the reaction system (Figure 4B). It is worth noting that, when the volume fraction of ethanol is 10%, the relative activity of the Tris-Cu nanozyme and laccase is 80.5% and 27.9%, respectively. When the volume fraction of ethanol is 20%, the relative activity of the Tris-Cu nanozyme and laccase is 56.8% and 14.4%, respectively. In addition, as illustrated in Figure 4C, the Tris-Cu nanozyme and laccase maintained approximately 91% and 75% of their relative activity, respectively, after being stored at 2 °C and pH 6.0 for eight days. These results indicate that the Tris-Cu nanozyme exhibits excellent robustness compared to natural laccase.

### 3.4. Mechanism Investigation

The reaction process of 2,4-DP catalyzed by the AP-Cu nanozyme was investigated (Figure 4D–F). The experimental result (Figure 4D) shows that the addition of the hydroxyl radical scavenger IPA has no effect on the catalytic activity of the Tris-Cu nanozyme, which indicates that •OH does not participate in the catalytic reaction. However, the activity of the Tris-Cu nanozyme is inhibited by the superoxide anion radical scavenger 1,4-benzoquinone, suggesting that superoxide anion radicals play an important role in this reaction. To further investigate the catalytic mechanism of the Tris-Cu, the changes in the absorbance of the reaction system (Tris-Cu nanozyme, 2,4-DP, and 4-AP) under N_2_ and O_2_ saturation conditions were monitored (Figure 4E). The results indicate that oxygen is required in the reaction process. The physiologically accessible Cu(I)/Cu(II)-coupled redox potential varies significantly depending on the ligand environment; for example, copper ion complexes have a wide reduction potential (from −1.5 V to +1.3 V, compared to standard hydrogen electrodes) during the single-electron oxidation of oxygen molecules [36]. It can be speculated that Cu(I)/Cu(II) in the Tris-Cu nanozyme is the catalytic center for the oxidation of phenolic compounds. In summary, the catalytic mechanism of the Tris-Cu nanozyme is proposed as follows. As shown in Figure 4F, first, the substrate (2,4-DP) is bound to the Tris-Cu nanozyme. Then, 2,4-DP is oxidized by Tris-Cu, loses electrons, and is coupled with the color developer 4-AP to form a rose-red oxidation product (Quinone-imine, QI). During this process, the electrons released from 2,4-DP are taken up by Cu(II) in Tris-Cu, and Cu(II) is converted to Cu(I). Then, O_2_ in the reaction system is combined with Cu(I) in Tris-Cu, Cu(I) is re-oxidized to Cu(II), and O_2_ is converted to water. Thus, the catalytic cycle of the nanozyme is realized. The catalytic cycle of the Tris-Cu nanozyme is like that of the reported laccase and copper-containing laccase mimics [20,27,28,37].

### 3.5. Quantitative Detection of Phenolic Compounds

**Figure 5:**
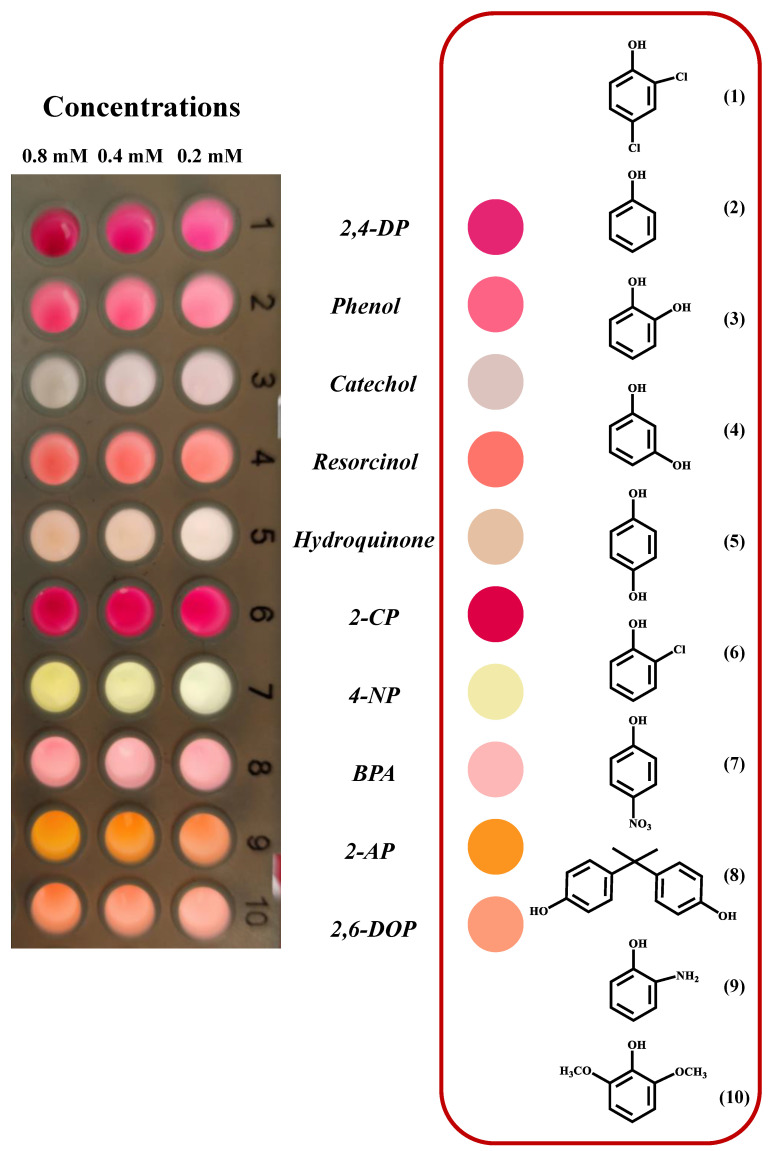
Different phenolic compounds were oxidized by the Tris-Cu nanozyme. (**1**) 2,4-dichlorophenol (2,4-DP), (**2**) phenol, (**3**) catechol, (**4**) resorcinol, (**5**) hydroquinone, (**6**) 2-chlorophenol (2-CP), (**7**) 4-nitrophenol (4-NP), (**8**) bisphenol A (BPA), (**9**) 2-aminophenol (2-AP), (**10**) 2,6-dimethoxyphenol (2,6-DOP); the concentrations of all phenolic compounds are 0.2 mM.

**Figure 6:**
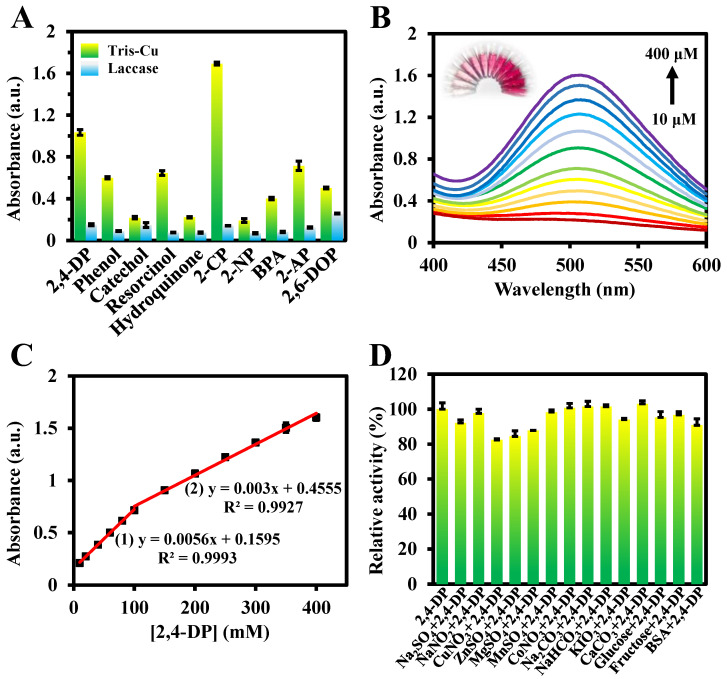
Catalytic performance of the Tris-Cu nanozyme and laccase on different phenolic compounds (0.2 mM) (**A**). UV–Vis spectra of the Tris-Cu solutions in the presence of 10–400 μM 2,4-DP in 10 mM NaAc (pH 6.0) and the corresponding solution photographs (**B**). The relationship between the 2,4-DP concentration and the corresponding absorption intensity at 510 nm (**C**). Effects of different interfering substances (1 mg/mL) on the laccase-like activity of the Tris-Cu nanozyme (**D**).

Due to its broad substrate specificity, laccase can catalyze the oxidation of different phenolics. To test the substrate diversity of the Tris-Cu nanozyme and to perform the simple identification of phenolic compounds before quantitation, the catalytic activity of the Tris-Cu nanozyme on different phenolic compounds was studied and the color of the corresponding oxidation products was observed. As illustrated in Figure 5, Figure S6 and Figure 6A, the Tris-Cu nanozyme shows good catalytic activity against ten phenolic compounds, including 2,4-DP, phenol, catechol, resorcinol, hydroquinone, 2-CP, 4-nitrophenol (4-NP), BPA, 2-aminophenol (2-AP), and 2,6-DOP. We found that the colors of the oxidation products of different phenolic compounds were different, and the color gradually deepened with the increase in the concentrations (0.2, 0.4, and 0.8 mM) of the substrates. The possible reasons for the Tris-Cu nanozyme exhibiting different catalytic activities against different phenolic compounds are as follows. (1) The Tris-Cu nanozyme has different affinities for different phenolic compounds. (2) During the process of binding to the nanozyme catalytic center, the probability and difficulty of collisions between different phenolic compounds and the nanozyme catalytic center differ, due to the influence of steric hindrance and configuration. (3) Different substituents have different effects on the electron cloud of the benzene ring (the electron induction or conjugation effect) and then have different effects on the electron cloud density of the phenolic hydroxyl, which ultimately leads to the difference in the difficulty of the electron loss of the phenolic hydroxyl group during the oxidation process. In addition, the results shown in Figure S7 indicate that the Tris-Cu nanozyme has excellent selectivity for phenolic compounds. Therefore, based on the above results, the visual identification of phenolic compounds can be preliminarily realized.

Then, a colorimetric sensor was constructed for the rapid detection of 2,4-DP based on the efficient catalytic activity of the Tris-Cu nanozyme. As shown in Figure 6B,C, the color of the reaction system gradually deepens with the increase in the 2,4-DP concentration, which is also reflected in the absorbance of the reaction system. The 2,4-DP concentrations between 10 μM–100 μM and 100 μM–400 μM have good linear correlations with the absorbance at 510 nm, with the linear regression equations of *A* = 0.0056 [*2,4-DP*] + 0.1595 (R^2^ = 0.9993) and *A* = 0.003 [*2,4-DP*] + 0.4555 (R^2^ = 0.9927), respectively. The difference in the linear slopes of the two 2,4-DP concentration ranges may be due to the different reaction states of the nanozymes at high or low concentrations of 2,4-DP. The LOD is 2.40 μM. Compared with previously reported methods, this colorimetric method has the advantages of a wide detection range, simple operations, and good sensitivity (Table 2).

In addition, to evaluate the colorimetric method in practical applications, different interfering substances are added to the catalytic system to investigate their influence on the relative activity of the Tris-Cu nanozyme. The results show that the effect of the investigated interfering substances on the catalytic activity of the Tris-Cu nanozyme is negligible except for three metal ions, Cu^2+^, Zn^2+^, and Mg^2+^ (Figure 6D). However, since the concentrations of Cu^2+^, Zn^2+^, and Mg^2+^ used in the experiment are much higher than those present in the actual sample, their effect on the activity of the Tris-Cu nanozyme can be ignored. Subsequently, the established method was applied to the detection of 2,4-DP in tap and lake water samples. Different final concentrations of 2,4-DP (10 mM, 100 mM, and 200 mM) were spiked into three groups of tap and lake water samples, and the recovery and RSD values were determined. As shown in Table 3, the recovery and RSD of the established colorimetric method are satisfactory, which indicates that the Tris-Cu nanozyme has great potential in practical applications. However, since Cl ions can promote the catalytic activity of the Tris-Cu nanozyme, the linear regression equation needs to be reconstructed in the analysis of actual samples containing Cl ions, such as seawater.

### 3.6. Removal of Phenolic Contaminants by the Tris-Cu Nanozyme

**Figure 7:**
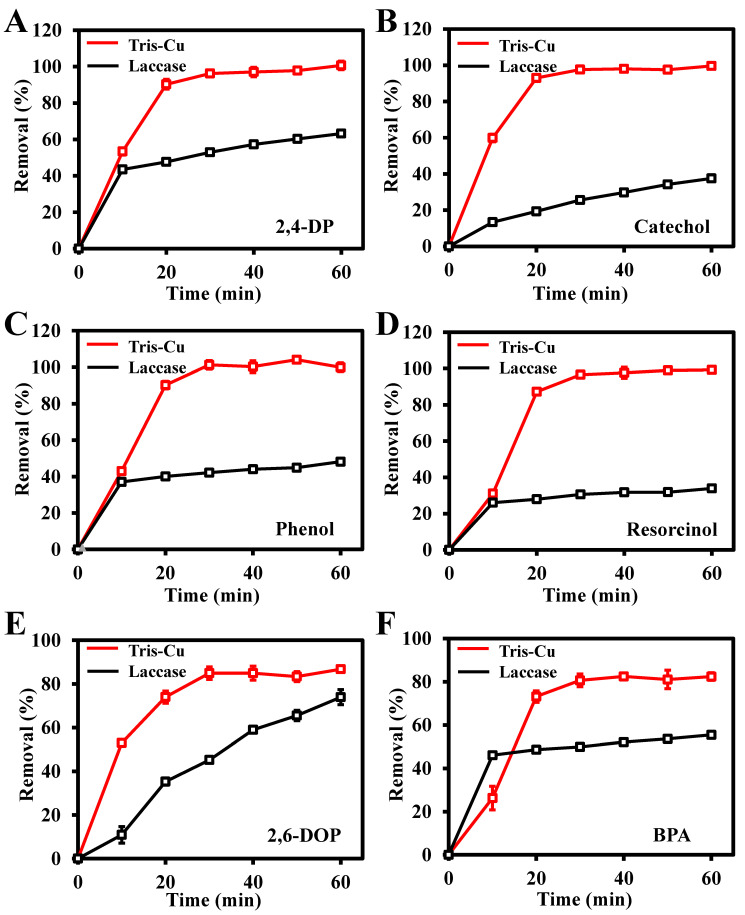
Removal efficiency of different phenolic compounds by the Tris-Cu nanozyme and laccase over time. 2,4-DP (**A**), catechol (**B**), phenol (**C**), resorcinol (**D**), 2,6-DOP (**E**), BPA (**F**); the concentrations of all phenolic compounds are 0.1 mM.

To study the removal efficiency of the Tris-Cu nanozyme on phenolic compounds, 2,4-DP, 2-CP, phenol, resorcinol, 2,6-DOP, and BPA were selected as the study objects. As illustrated in Figure S8, the content of residual phenolic compounds in the sample was determined using the 4-aminoantipyrine spectrophotometry method. Figure 7 shows that the Tris-Cu nanozyme has higher removal efficiency for these six phenolic compounds than natural laccase. Among them, the removal efficiencies of the Tris-Cu nanozyme and natural laccase for these six phenolic compounds (2,4-DP, 2-CP, phenol, resorcinol, 2,6-DOP, and BPA) within one hour are 100%, 100%, 100%, 100%, 87%, and 81%, and 63%, 38%, 48%, 35%, 55%, and 55%, respectively. Compared with natural laccase, the Tris-Cu nanozyme shows a higher removal efficiency and reaction rate in phenolic compound degradation, which further proves the superior catalytic performance of the Tris-Cu nanozyme, as it has a stronger substrate affinity (*K_m_*), a higher catalytic activity (*V_max_*), and a higher turnover number (*K_cat_*) than natural laccase. In addition, the prepared Tris-Cu nanozyme has outstanding advantages in the degradation of phenolic compounds compared to the other catalysts (Tables S3–S8). To further evaluate the application potential of the Tris-Cu nanozyme, 2,4-DP was selected as the target substrate for the degradation study in simulated sewage. As shown in Figure S9, the reaction kinetics of degradation of 2,4-DP by the Tris-Cu nanozyme were investigated; the calculated reaction rate constant (*k*_0_) is 0.2304 min^−1^ and the degradation efficiency of the Tris-Cu nanozyme to 2,4-DP (0.1 mM or 16.3 mg/L) reaches 76.36% at 7 min. Compared to other catalytic methods, the method for the degradation of 2,4-DP based on the highly efficient laccase-like activity of the Tris-Cu nanozymes has the advantages of a fast reaction rate and simple operations (Table S9). Since Cl ions can enhance the catalytic activity of the Tris-Cu nanozyme, it has application potential in degrading phenolic compounds in seawater.

## 4. Conclusions

A Tris-Cu nanozyme with efficient laccase-like activity is prepared for the first time via a simple and rapid synthesis method. Like natural laccase, the multivalent Cu ions in Tris-Cu play a key role in the catalytic process. The acid and alkali resistance, robustness, catalytic activity, and substrate affinity of the Tris-Cu nanozyme are obviously better than those of natural laccase, indicating its potential in practical applications. Based on the efficient catalytic activity of the Tris-Cu nanozyme, a colorimetric sensing method for the preliminary identification and detection of phenolic compounds was successfully constructed; it has the characteristics of a wide detection range, simple operations, fast speeds, and visual detection. Furthermore, tests in actual samples verify that the method exhibits good accuracy. In the degradation experiments with phenolic compounds, the Tris-Cu nanozyme has efficient removal efficiency and a faster reaction rate than natural laccase and some other nanozymes, which indicates that the Tris-Cu nanozyme has application value in environmental remediation. This study not only expands the methods used for the colorimetric detection and catalytic degradation of phenolic compounds but also has reference significance for the design and research of novel laccase mimics.

## Figures and Tables

**Table 1:** Kinetic parameters of 2,4-DP and 4-AP reactions catalyzed by Tris-Cu, laccase, and various laccase mimics.

Catalysts	*ρ_e_* (g L^−1^)	[*E*] (mM)	*K_m_* (mM)	*V_max_* (μM min^−1^)	*K_cat_* (min^−1^)	*K_cat_*/*K_m_* (mM^−1^ min^−1^)	Ref.
Laccase	0.10	1.55 × 10^−3^	0.41	6.41	4.13	10.07	[26]
CH-Cu	0.10	3.83 × 10^−7^	0.42	7.32	1.91 × 10^4^	4.55 × 10^4^	
BSA-Cu	-	1.5 × 10^−3^	0.115	4.00	2.66	23.19	[27]
Cu FMA	5.00	1.98 × 10^−9^	0.45	3.43	1.73 × 10^6^	3.84 × 10^6^	[28]
Ce-MOF-808	0.10	6.27 × 10^−2^	0.13	2.22	0.035	0.27	[29]
MOF-His-Cu	0.06	-	2.21	69.6	0.414	0.19	[30]
CA-Cu	0.05	-	0.232	11.22	0.1518	0.65	[31]
Tris-Cu	0.44	9.98 × 10^−10^	0.18	15.62	1.57 × 10^7^	8.72 × 10^7^	This work

CH: cysteine (Cys)-histidine (His) dipeptide; BSA: bovine serum albumin; Cu FMA: copper fumarate; His: histidine; CA: cyanuric acid; Tris: Tris(hydroxymethyl)aminomethane.

**Table 2:** Comparison of different methods for the detection of 2,4-DP.

Methods	Material System	Linear Rang (μM)	LOD (μM)	Ref.
Chemiluminescence	CdTe quantum dot	0.36–36	0.13	[38]
Electrochemical	Mb-AG/GCE	12.5–208	2.06	[39]
Electrochemical	Laccase/PVA/F108/Au NPs/GCE	1.0–25.0	2.70	[40]
Electrochemical	Gr/PPyNT/SrCuO_2_/lac	1–50	0.18	[41]
Electrochemical	Cu-MOF	4–100	1.10	[42]
UV–Vis	Cu-MeIm nanozyme	200–2000	34	[37]
UV–Vis	Cu-Adenine nanozyme	1–100	0.472	[33]
UV–Vis	Tris-Cu nanozyme	10–400	2.40	This work

Mb: myoglobin; AG: agarose hydrogel; GCE: glassy carbon electrode; PVA: polyvinyl alcohol; F108: PEO–PPO–PEO; PPyNT: polypyrrole nanotube; lac: laccase; MeIm: 2-Methylimidazole.

**Table 3:** Detection of 2,4-DP in tap and lake water samples using the developed colorimetric method (*n* = 3).

Actual Samples		Added (μM)	Detected (μM)	Recovery (%)	RSD (%)
Tap water	Sample 1	0	—	—	1.71
	Sample 2	10	9.95 ± 0.51	99.46	4.99
	Sample 3	100	104.43 ± 1.81	104.43	1.73
	Sample 4	400	427.89 ± 2.72	106.97	0.64
Lake	Sample 1	0	—	—	1.02
	Sample 2	10	9.94 ± 0.46	99.40	4.62
	Sample 3	100	96.9 ± 0.91	96.90	0.94
	Sample 4	400	409.44 ± 2.41	102.36	0.59

## Data Availability

Data are contained within the article or Supplementary Materials.

## Supplementary Materials

The following supporting information can be downloaded at: https://www.mdpi.com/article/10.3390/s23198137/s1, Figure S1: XPS analysis of AP-Cu nanozyme: C1s (A); O 1s (B); Figure S2: Effect of different feeding ratios of Tris to copper ions (A) and the concentration of copper ion (B) on the activity of the Tris-Cu nanozyme; Figure S3: The laccase-like activity of the Tris-Cu nanozyme in different buffers; Figure S4: The Zeta potential of the Tris-Cu nanozyme in the NaAc buffer at different pH values; Figure S5: The relationship between the 2,4-DP concentration and the corresponding absorption intensity at 510 nm (A); Double-reciprocal plots: 2,4-DP, 0.04–0.2 mM; 4-AP, 0.2 mM (B); Figure S6: Catalytic activity of the Tris-Cu nanozyme for different phenolic compounds. (A, 2,4-DP; B, phenol; C, catechol; D, resorcinol; E, hydroquinone; F, 2-CP; G, 4-nitrophenol (4-NP); H, BPA; I, 2-aminophenol (2-AP); J, 2,6-DOP); Figure S7: Catalytic activity of the Tris-Cu nanozyme for different phenol analogues; Figure S8: Determination of phenolic compounds via 4-amino-antipyrine spectrophotometry. The liner relationship between the phenolic compound’s concentration and its corresponding absorption intensity at the maximum absorption wavelength. (A, 2,4-DP; B, 2-CP; C, phenol; D, resorcinol; E, 2,6-DOP; F, BPA; 0–100 μM); Figure S9: (A) Evolution of 2,4-DP concentration over time; (B) First-order kinetic plot for 2,4-DP degradation. (2,4-DP concentration = 0.1 mM); Table S1: Atomic ratios of various elements in Tris-Cu nanozymes (XPS); Table S2: Assigned details of the FT-IR spectra of Tris-Cu nanozymes, Tris and CuSO_4_·5H_2_O; Table S3: Comparison of the degradation efficiency of 2,4-dichlorophenol using different methods; Table S4: Comparison of the degradation efficiency of 2-chlorophenol using different methods; Table S5: Comparison of the degradation efficiency of phenol using different methods; Table S6: Comparison of the degradation efficiency of resorcinol using different methods; Table S7: Comparison of the degradation efficiency of 2,6-dimethoxyphenol using different methods; Table S8: Comparison of the degradation efficiency of bisphenol A using different methods; Table S9: Comparison of the degradation rate constant of 2,4-dichlorophenol using different methods. References [43,44,45,46,47,48,49,50,51,52,53,54,55,56,57,58,59,60,61,62,63,64,65,66] are cited in the supplementary materials.
