# Supplementary material for: Tris-Copper Nanozyme as a Novel Laccase Mimic for the Detection and Degradation of Phenolic Compounds
Source: Sensors (Basel). 2023 Sep 28;23(19):8137. doi: 10.3390/s23198137 (PMC10575388; doi:10.3390/s23198137)
Supplement: Supplementary file 1 [file sensors-23-08137-s001.zip › sensors-2620263-supplementary.pdf]

Supplementary Materials

Tris-Copper Nanozyme as a Novel Laccase Mimic for the Detection and Degradation of Phenolic Compounds

Tong-Qing Chai, Jia-Li Wang, Guo-Ying Chen, Ling-Xiao Chen and Feng-Qing Yang *

School of Chemistry and Chemical Engineering, Chongqing University, Chongqing 401331, China; 20175531@cqu.edu.cn (T.-Q.C.); 202118021010@cqu.edu.cn (J.-L.W.); 20221801017@stu.cqu.edu.cn (G.-Y.C.); 20175534@cqu.edu.cn (L.-X.C.)

* Correspondence: fengqingyang@cqu.edu.cn; Tel.: +86-13617650637

Table of Contents

Supplementary methods

1. Chemicals and reagents

2. Instrumentation

3. The 4-aminoantipyrine spectrophotometry

4. The preparation of simulate sewage

5. The method of calculating the relative activity

6. The method of calculating the limit of detection

Supplementary figures

Fig. S1. XPS analysis of AP-Cu nanozyme: C1s (**A**); O 1s (**B**).

Fig. S2. Effect of different feeding ratios of Tris to copper ions (**A**) and the concentration of copper ion (**B**) on the activity of Tris-Cu nanozyme.

Fig. S3. The laccase-like activity of Tris-Cu nanozyme in different buffers.

Fig. S4. The Zeta potential of Tris-Cu nanozyme in NaAc buffer at different pH.

Fig. S5. The relationship between 2,4-DP concentration and the corresponding absorption intensity at 510 nm (**A**); Double-reciprocal plots: 2,4-DP, 0.04–0.2 mM; 4-AP, 0.2 mM (**B**).

Fig. S6. Catalytic activity of Tris-Cu nanozyme for different phenolic compounds. (**A**, 2,4-DP; **B**, phenol; **C**, catechol; **D**, resorcinol; **E**, hydroquinone; **F**, 2-CP; **G**, 4-nitrophenol (4-NP); **H**, BPA; **I**, 2-aminophenol (2-AP); **J**, 2,6-DOP).

Fig. S7. Catalytic activity of Tris-Cu nanozyme for different phenol analogues.

Fig. S8. Determination of phenolic compounds by 4-amino-antipyrine spectrophotometry. The liner relationship between phenolic compound's concentration and its corresponding absorption intensity at the maximum absorption wavelength. (**A**, 2,4-DP; **B**, 2-CP; **C**, phenol; **D**, resorcinol; **E**, 2,6-DOP; **F**, BPA; 0–100 μM).

Fig. S9. (**A**) Evolution of 2,4-DP concentration over time; (**B**) First-order kinetic plot for 2,4-DP degradation. (2,4-DP concentration = 0.1 mM).

Supplementary tables

Table S1. Atomic ratios of various elements in Tris-Cu nanozymes (XPS)

Table S2. Assigned details of FT-IR spectra of Tris-Cu nanozymes, Tris and $CuSO_4 \cdot 5H_2O$

Table S3. Comparison of degradation efficiency of 2,4-dichlorophenol by different methods

Table S4. Comparison of degradation efficiency of 2-chlorophenol by different methods

Table S5. Comparison of degradation efficiency of phenol by different methods

Table S6. Comparison of degradation efficiency of resorcinol by different methods

Table S7. Comparison of degradation efficiency of 2,6-dimethoxyphenol by different methods

Table S8. Comparison of degradation efficiency of bisphenol A by different methods

Table S9. Comparison of degradation rate constant of 2,4-dichlorophenol by different methods

Supplementary methods

1. Chemicals and reagents

Tris(hydroxymethyl)methyl aminomethane (Tris, AR) and bovine albumin (BSA, Molecular Biology Grade) were purchased from BBI Life Sciences Co., Ltd. (Shanghai, China) (http://www.bbi-lifesciences.com/). Copper sulfate pentahydrate ($CuSO_4 \cdot 5H_2O$, AR), 2-morpholinoethanesulfonic acid (MES, 98%), N-(2-hydroxyethyl) piperazine-N'-2-ethanesulfonic acid, sodium salt (HEPES, 99.5%), 2,4-dichlorophenol (2,4-DP, 98%), 1,4-benzoquinone (AR), potassium ferricyanide ($K_3FeCN)_6$, AR), and bisphenol A (BPA, AR) were purchased from Meryer Chemical Technology Co., Ltd. (Shanghai, China) (https://www.meryer.com/). Sodium acetate anhydrous (NaAc, AR), methylbenzene (AR), acetic acid (HAc, AR), and phenol (AR) were purchased from Chongqing Chuandong Chemical (Group) Co., Ltd. (Chongqing, China) (http://www.cd1958.com/). Sodium chloride (NaCl, AR), sodium sulphate (Na_2SO_4, AR), sodium nitrite ($NaNO_2$, AR), copper(II) nitrate hydrate ($Cu(NO_3)_2 \cdot 5H_2O$, AR), zinc sulfate heptahydrate ($ZnSO_4 \cdot 7H_2O$, AR), magnesium sulfate heptahydrate ($MgSO_4 \cdot 7H_2O$, AR), manganese (II) sulfate monohydrate ($MnSO_4 \cdot H_2O$, AR), ammonium bicarbonate (NH_4HCO_3, AR), potassium dihydrogen phosphate (KH_2PO_4, AR), potassium chloride (KCl, AR), hydrochloric acid (HCl, AR), phosphoric acid (H_3PO_4, AR), sodium bicarbonate ($NaHCO_3$, AR), benzoic acid (AR), 4-nitrobenzaldehyde (98%), 4-aminobenzoic acid (AR), ethanol (AR), and isopropanol (IPA, AR) were purchased from Chengdu Chron Chemicals Co., Ltd. (Chengdu, China) (http://www.chronchem.com/cn/). Sodium dihydrogen phosphate anhydrous (NaH_2PO_4,

AR), sodium hydroxide (NaOH, AR), sodium carbonate (Na_2CO_3, AR)， 4-methoxybenzoic acid (99%), resorcinol (99%), and 2-chlorophenol (99%) were purchased from Shanghai Titan Technology Co., Ltd. (Shanghai, China) (https://www.titansci.com). The 4-aminoantipyrine (4-AP, 98%), hydroquinone (AR), cobaltous nitrate hexahydrate ($Co(NO_3)_2 \cdot 6H_2O$), calcium carbonate ($CaCO_3$, AR), fructose (99%), and 2,6-dimethoxyphenol (2,6-DOP, 98%) were purchased from Shanghai Macklin Biochemical Co., Ltd. (Shanghai, China) (http://www.macklin.cn/). 2-Aminophenol (2-AP, 99%) and potassium iodate (KIO_3, AR) were purchased from Beijing Mreda Technology Co., Ltd. (Beijing, China) (https://www.mreda.com.cn/). Laccase (200 U/g) from *Aspergillus* was purchased from Beijing Solarbio Technology Co., Ltd. (Beijing, China) (http://www.solarbio.jqw.com/). 4-Nitrophenol (4-NP, AR) and catechol (AR) were purchased from Aladdin Reagent Co., Ltd. (Shanghai, China) (https://www.aladdin-e.com/). Albumin human serum (Sigma A-1653) was purchased from Beijing Biodee Biological Co., Ltd. (Beijing, China) (https://www.biodee.net/). L-tyrosine (AR) was purchased from Chengdu Huaxia Chemical Reagent Co., Ltd. (Chengdu, China) (https://www.biochemsafebuy.com/). D-(+)-glucose (AR) was purchased from Shanghai YuanYe Biological Technology Co., Ltd. (Shanghai, China) (http://yuanyebio.bioon.com.cn/). Calcium chloride ($CaCl_2$, AR) was purchased from Tianjin Damao Chemical Reagent Factory (Tianjin, China) (https://www.dmreagent.com). Benzyl alcohol (AR) was purchased from Shenyang Reagent No. 3 Factory (Shenyang, China) (https://www.11467.com/shenyang/co/76115.htm). All chemicals were used as

received without further purification. Water used for all the experiments was purified by a water purification system (ATSelem 1820A, Antesheng Environmental Protection Equipment Co., Ltd., Chongqing, China) (http://www.atshb.com/).

2. Instrumentation

Scanning electron microscopy (SEM) images were obtained using a field-emission scanning electron microscope (FESEM) (Quanta 650, FEI, Hillsboro, OR, https://www.fei.com) at 20 kV. Transmission electron microscopy (TEM) images and element distribution analysis were recorded using a JEM 2100 electron microscope (JEOL Ltd. Tokyo, Japan, https://www.jeol.co.jp/en/) working at 200 kV, which is equipped with energy dispersive X-ray spectrometer (EDX). X-ray diffraction (XRD) patterns were obtained using X'pert Powder diffractometer (Malvern Panalytical Ltd., Netherlands, https://www.malvernpanalytical.com/en/) with secondary beam graphite monochromated Cu Kα radiation. X-ray photoelectron spectrometry (XPS) was recorded on a PHI5000 Versaprobe system using monochromatic Al Ka radiation (1486.6 eV), and the obtained binding energies were referenced to the C 1s line set at 284.8 eV (Thermo Fisher Scientific Ltd, UK, https://www.thermofisher.cn/). Fourier transform infrared spectra (FT-IR) were taken on a Nicolet iS50 spectrometer (Thermo Fisher Scientific, USA, https://www.thermofisher.cn) between 4000 cm^{-1} and 400 cm^{-1} in KBr media. Blast drying oven (DHG-9015A) was purchased from Shanghai Yiheng Scientific Instrument Co., Ltd. (Shanghai, China) (http://www.yihengchina.com/). Micro Pocket Centrifuge (HL-4K, 4000 r/min) was purchased from Shanghai Huxi Industrial Co., Ltd. (Shanghai, China) (http://www.huxishiye.com). Ultrasonic cleaner

(KS-3200B) was purchased from Kunshan Jielimei Ultrasonic Instrument Co., Ltd. (Hangzhou, China) (http://www.ks-jlmcsyq.com). Precision balance (ATX124) was purchased from Shimadzu. (Japan) (http://www.shimadzu.com). UV-Vis spectrophotometer (UV-8000S) was purchased from Shanghai Metash Instruments Co., Ltd. (Shanghai, China) (http://www.metash.com).

3. The 4-aminoantipyrine spectrophotometry

The content of phenolic compounds in the sample was determined by the 4-aminoantipyrine spectrophotometry [1-3]. In brief, 100 μL of potassium ferricyanide (25 mM) and 100 μL of 4-aminoantipyrine (5 mM) were added to PBS buffer containing different concentrations of phenolic compounds (total 500 μL). After 5 min of complete color formation of the reaction mixture, the absorbance at the maximum absorption wavelength was recorded. After that, these calibration curves between the absorbance value and the phenolic compounds' concentrations were calculated.

4. The preparation of simulate sewage

To prepare simulated sewage, glucose (1000 mg), NH_4HCO_3 (800 mg), KH_2PO_4 (40 mg), NaCl (500 mg), KCl (500 mg), $CuNO_3$ (50 mg), $MgSO_4$ (50 mg), and $CaCl_2$ (50 mg) are dissolved in 1 L of tap water. The theoretical chemical oxygen demand (COD), $NH4^+$-N, TN, and pH of simulated sewage are 1067 mg/L, 142 mg/L, 148 mg/L, and 7.58, respectively.

5. The method of calculating the relative activity

The value of the relative activity was calculated by the following formula.

$$\text{Relative activity (\%)} = A/A_{max} \times 100\% \quad (1)$$

Where A is the absorbance of the supernatant at 510 nm, A_{max} is the largest value of A.

6. The method of calculating the limit of detection

The limit of detections (LOD) is calculated as follows.

$$\text{LOD} = 3\sigma/b \quad (2)$$

Where σ is the standard deviation of 11 blank values (the deviation of the response value) and b is the slope of the calibration curve.

Supplementary figures

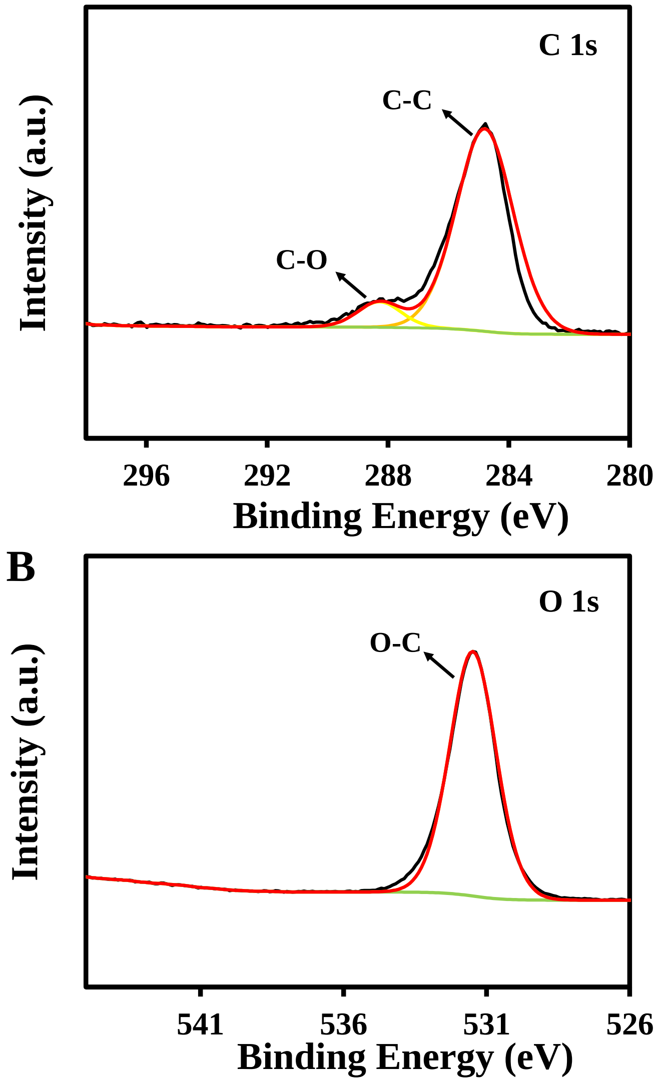

Figure S1. XPS analysis of AP-Cu nanozyme: C1s (**A**); O 1s (**B**).

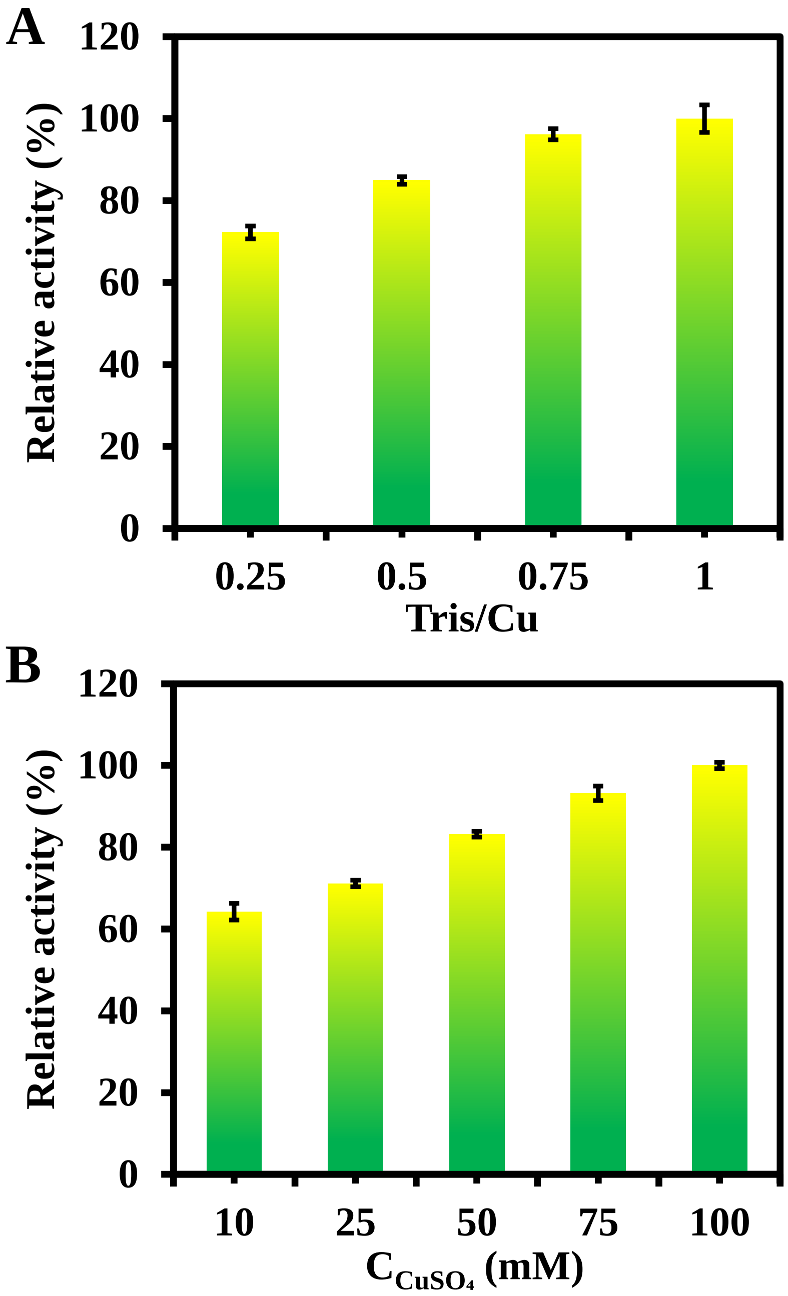

Figure S2. Effect of different feeding ratios of Tris to copper ions (**A**) and the concentration of copper ion (**B**) on the activity of Tris-Cu nanozyme.

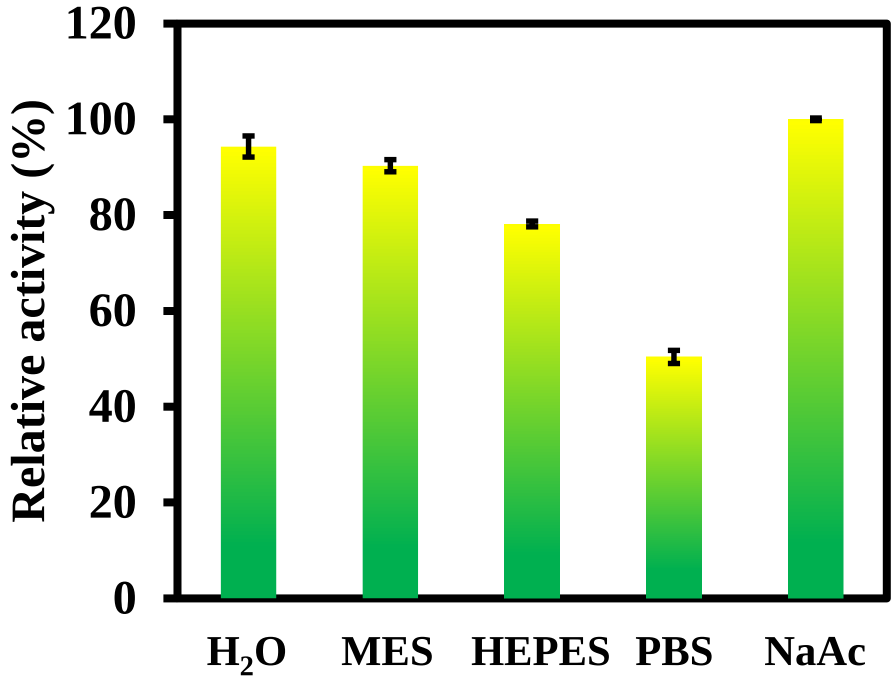

Figure S3. The laccase-like activity of Tris-Cu nanozyme in different buffers.

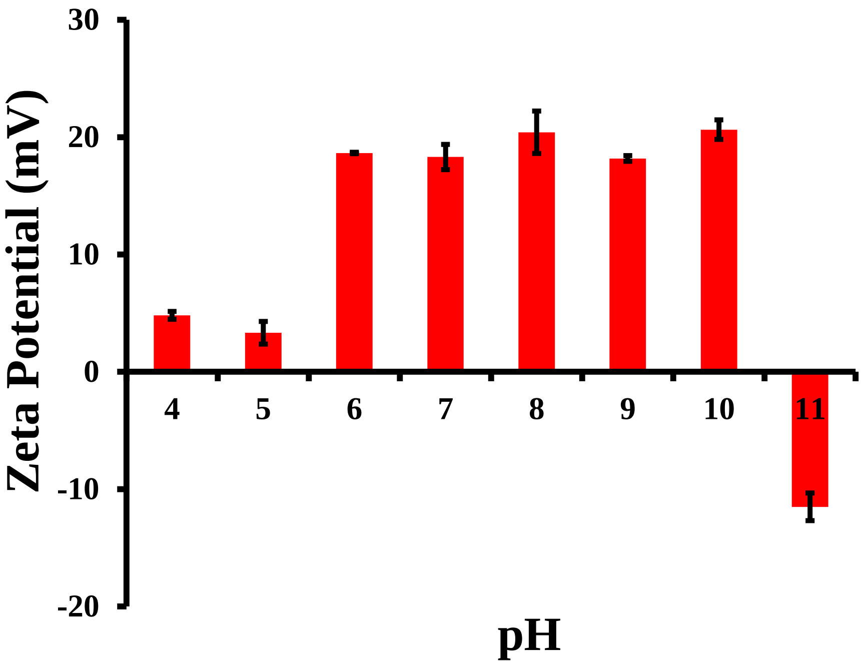

Figure S4. The Zeta potential of Tris-Cu nanozyme in NaAc buffer at different pH.

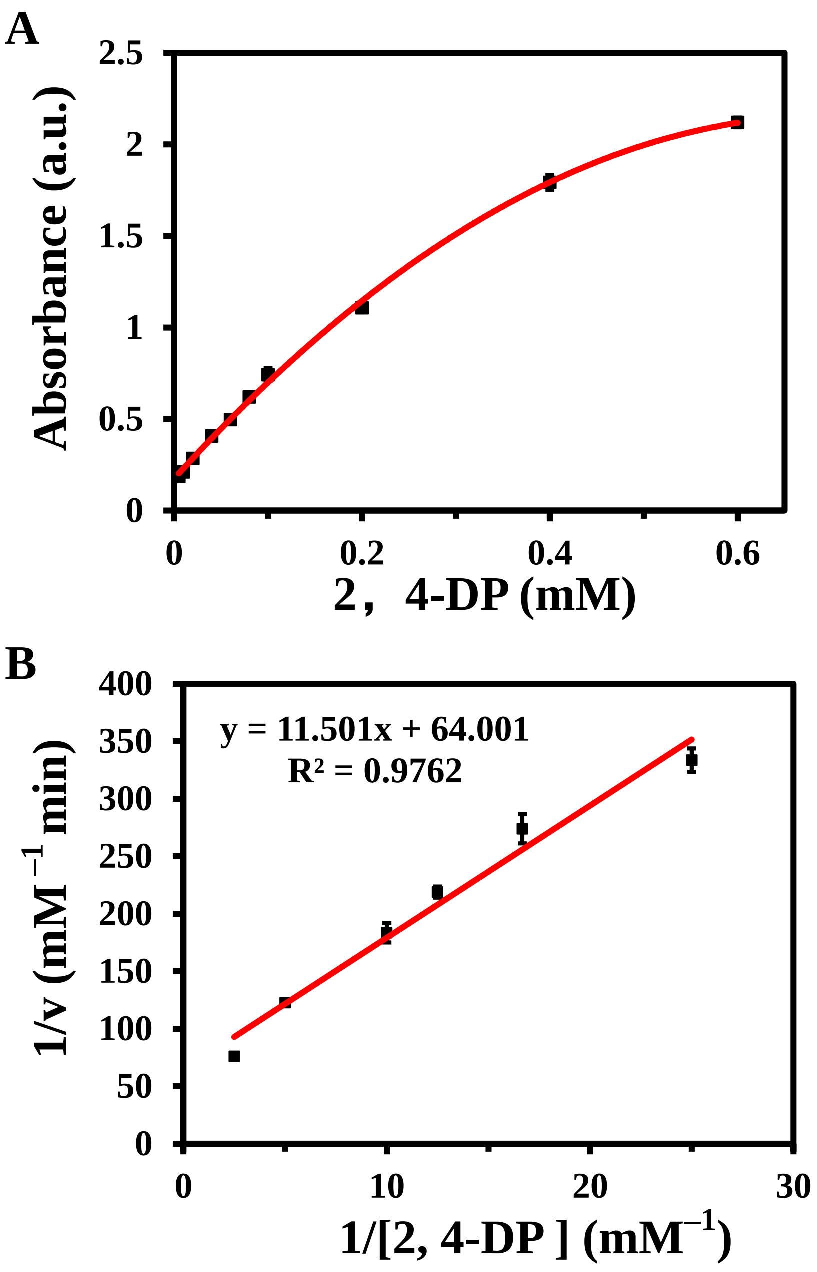

Figure S5. The relationship between 2,4-DP concentration and the corresponding absorption intensity at 510 nm (**A**); Double-reciprocal plots: 2,4-DP, 0.04–0.2 mM; 4-AP, 0.2 mM (**B**).

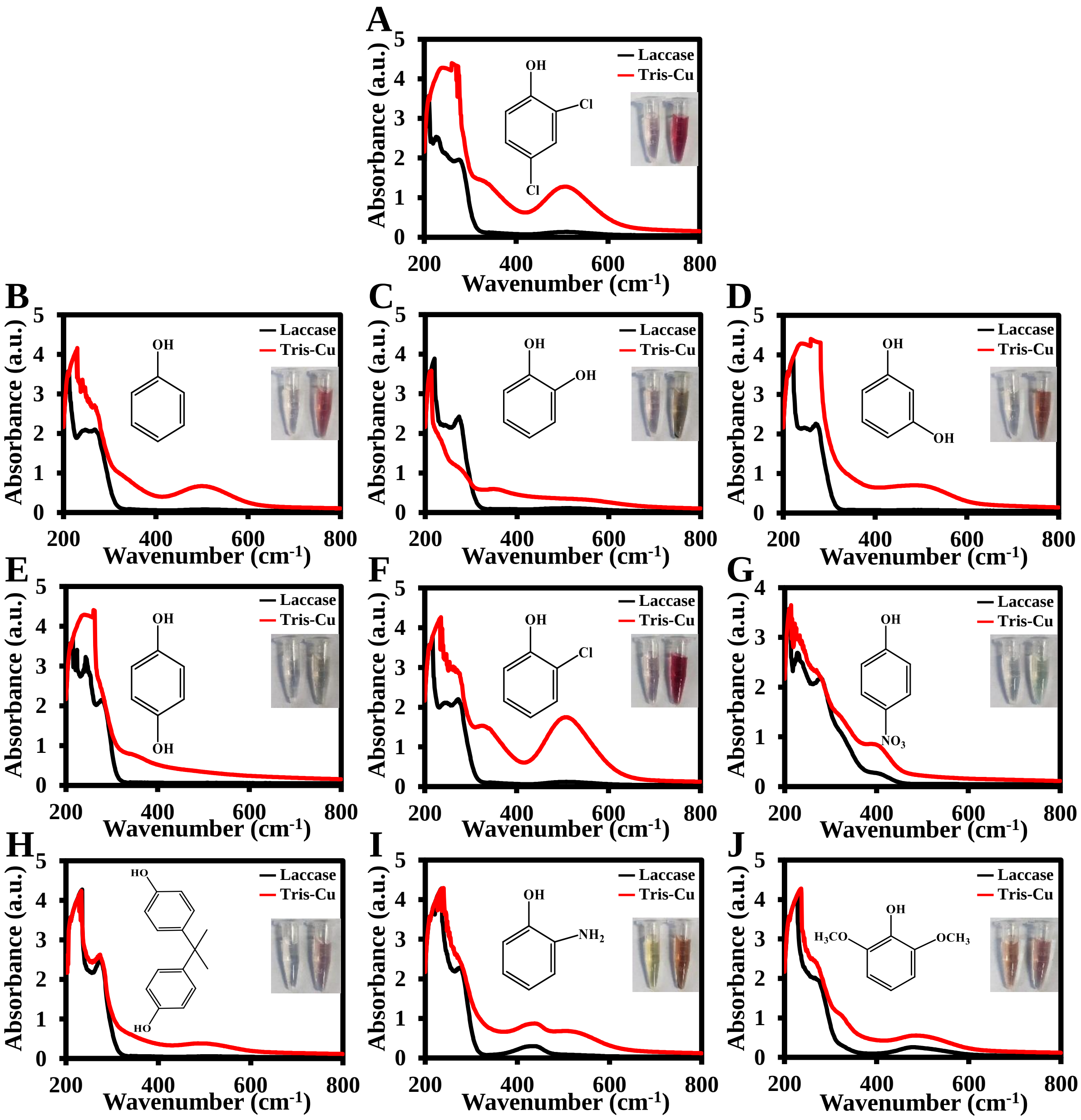

Figure S6. Catalytic activity of Tris-Cu nanozyme for different phenolic compounds. (**A**, 2,4-DP; **B**, phenol; **C**, catechol; **D**, resorcinol; **E**, hydroquinone; **F**, 2-CP; **G**, 4-nitrophenol (4-NP); **H**, BPA; **I**, 2-aminophenol (2-AP); **J**, 2,6-DOP).

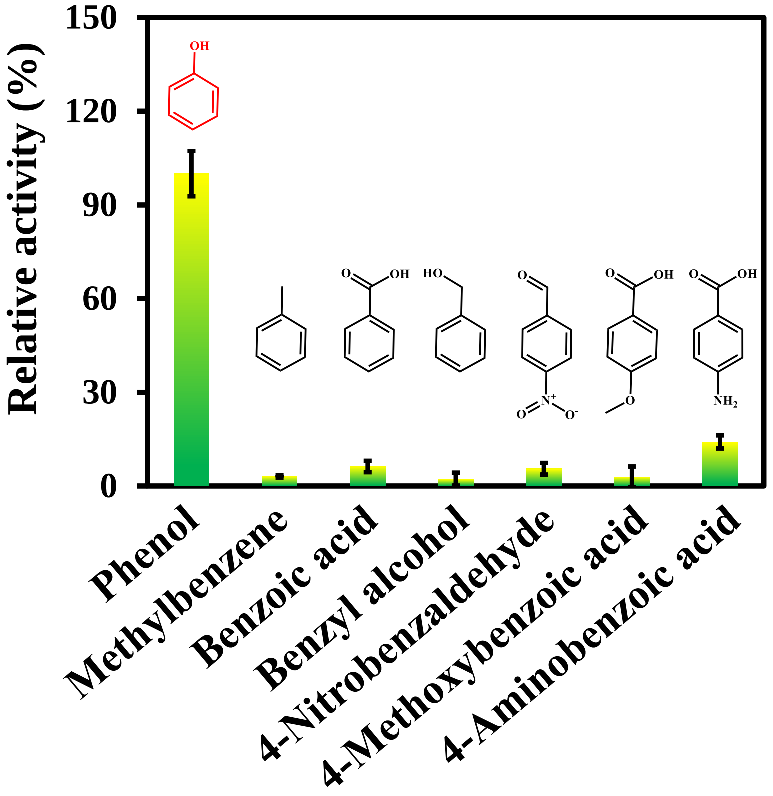

Figure S7. Catalytic activity of Tris-Cu nanozyme for different phenol analogues.

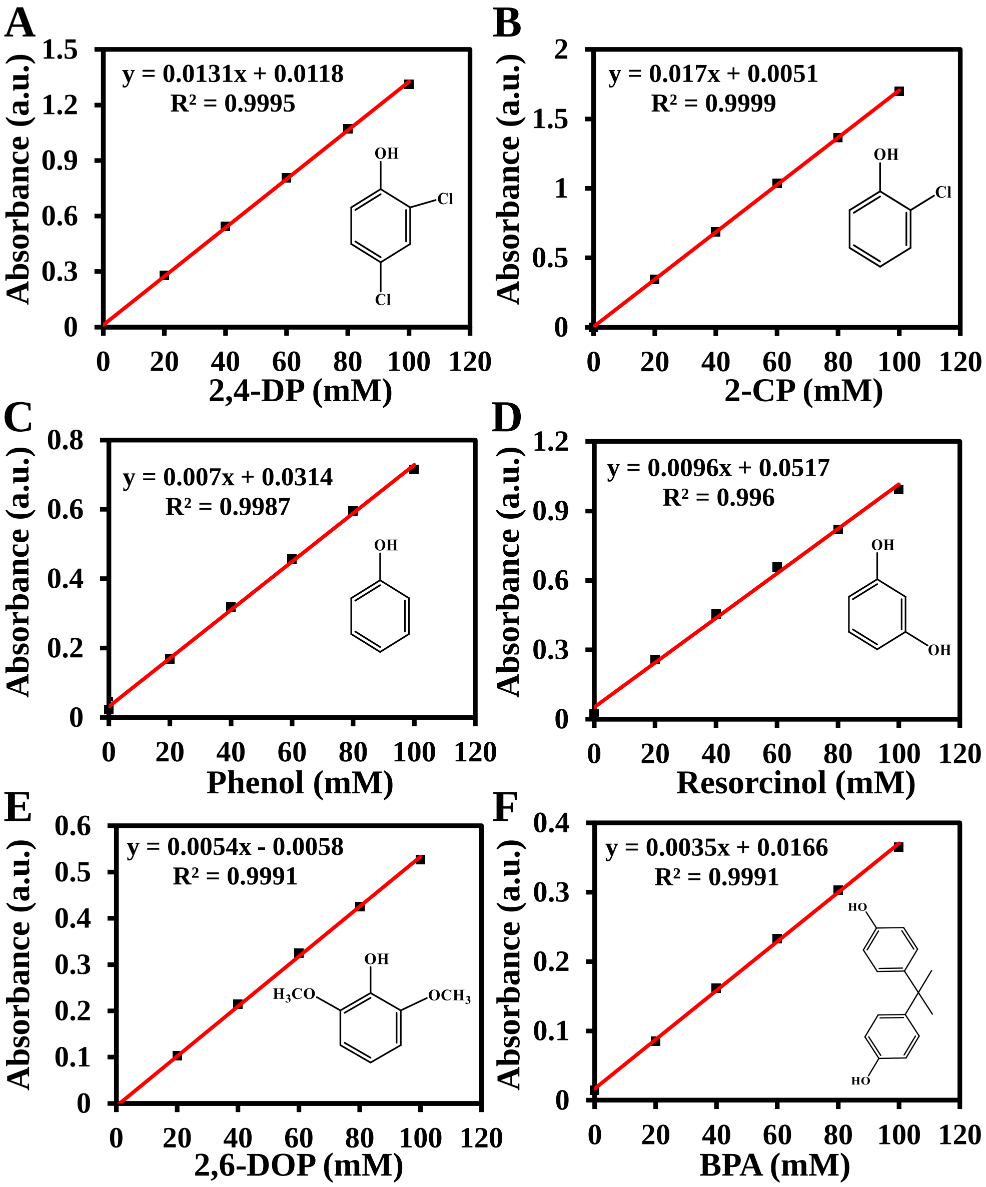

Figure S8. Determination of phenolic compounds by 4-aminoantipyrine spectrophotometry. The liner relationship between phenolic compound's concentration and its corresponding absorption intensity at the maximum absorption wavelength. (**A**, 2,4-DP; **B**, 2-CP; **C**, phenol; **D**, resorcinol; **E**, 2,6-DOP; **F**, BPA; 0–100 μM).

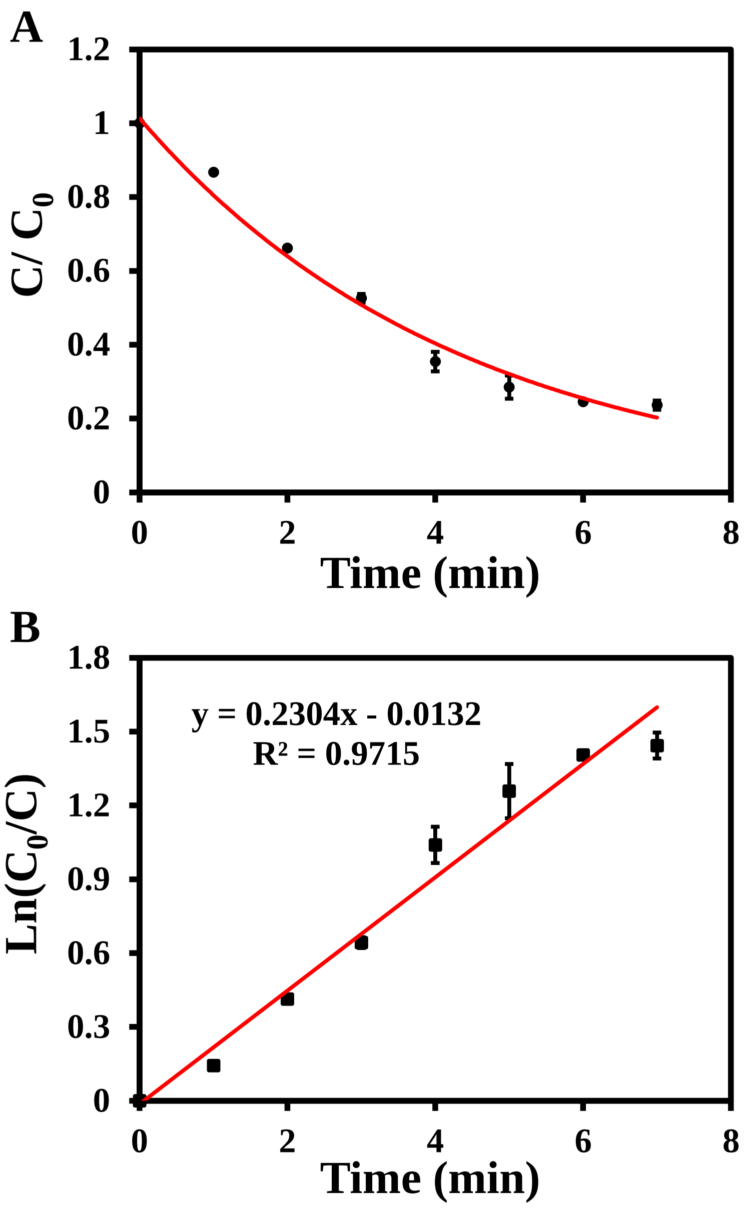

Figure S9. (**A**) Evolution of 2,4-DP concentration over time; (**B**) First-order kinetic plot for 2,4-DP degradation. (2,4-DP concentration = 0.1 mM).

Supplementary tables

Table S1. Atomic ratios of various elements in Tris-Cu nanozymes (XPS)

Element	Atomic (%)
C	35.31
Cu	14.98
N	4.54
O	45.17

Table S2. Assigned details of FT-IR spectra of Tris-Cu nanozymes, Tris and $CuSO_4 \cdot 5H_2O$

	**Tris-Cu**	**Tris**	**$CuSO_4 \cdot 5H_2O$**
ν_{OH}	3422 cm^{-1}	3348 cm^{-1}	3387 cm^{-1}
$\nu_{as\ NH_2}$	—	3201 cm^{-1}	—
$\nu_{s\ NH_2}$	—	2938 cm^{-1}	—
δ_{H_2O}	—	—	1623 cm^{-1}
δ_{NH_2}	—	1589 cm^{-1}	—
$\nu_{C\text{-}N}$	1110 cm^{-1}	1157 cm^{-1}	—
$\nu_{as\ SO_4^{2+}}$	—	—	1201, 1155 cm^{-1}
$\nu_{C\text{-}O}$	970 cm^{-1}	1018 cm^{-1}	—
$\delta_{as\ SO_4^{2+}}$	—	—	658 cm^{-1}
$\gamma_{C\text{-}O}$	617 cm^{-1}	630 cm^{-1}	—

ν: Stretching vibration; ν_{as}: Asymmetric stretching vibration; ν_s: Symmetrical stretching vibration; δ: Bending vibration; γ: Out-of-plane bending vibration; δ_{as}: Asymmetric deformation vibration.

Table S3. Comparison of degradation efficiency of 2,4-dichlorophenol by different methods

<table>
<tr><th>Degradation method</th><th>Catalyst</th><th>Catalyst concentration</th><th>Substrate concentration</th><th>Temperature</th><th>pH</th><th>Time</th><th>Degradation efficiency</th><th>Ref.</th></tr>
<tr><td>Catalytic degradation</td><td>CH-Cu nanozymes</td><td>0.1 g/L</td><td>100.0 mg/L</td><td>25 ℃</td><td>6.8</td><td>12 h</td><td>90%</td><td>[26]</td></tr>
<tr><td>Catalytic degradation</td><td>Imidazole-Cu nanozyme</td><td>0.1 g/L</td><td>100.0 mg/L</td><td>25 ℃</td><td>6.8</td><td>10 h</td><td>92%</td><td>[20]</td></tr>
<tr><td>Sonocatalytic degradation</td><td>Fe-TiO_2@Fe_3O_4 nanoparticles</td><td>0.4 g/L</td><td>20.0 mg/L</td><td>25 ℃</td><td>5.0</td><td>90 min</td><td>94%</td><td>[44]</td></tr>
<tr><td>Photodegradation</td><td>MIL-100(Fe) derivatives</td><td>0.75 g/L</td><td>100.0 mg/L</td><td>25 ℃</td><td>6.0–6.5</td><td>7 h</td><td>88%</td><td>[45]</td></tr>
<tr><td>Biodegradation</td><td>Aspergillus awamori cells</td><td>1×10^5 conidia / mL medium</td><td>3.0 g/L</td><td>30 °C</td><td>5.5</td><td>6 days</td><td>85%</td><td>[46]</td></tr>
<tr><td>Biodegradation</td><td>Laccase</td><td>2.2 mg/L (200 U/g)</td><td rowspan="2">16.3 mg/L</td><td rowspan="2">70 °C</td><td rowspan="2">6.0</td><td>20 min
60 min</td><td>48%
63%</td><td rowspan="2">This work</td></tr>
<tr><td>Catalytic degradation</td><td>Tris-Cu</td><td>0.44 mg/L</td><td>20 min
60 min</td><td>90%
100%</td></tr>
</table>

CH: Cysteine-histidine dipeptide.

Table S4. Comparison of degradation efficiency of 2-chlorophenol by different methods

Degradation method	Catalyst	Catalyst concentration	Substrate concentration	Temperature	pH	Time	Degradation efficiency	Ref.
Catalytic degradation	M/Z_x/PMS	0.1 g/L	100.0 mg/L	25 °C	9.0	10 min	90%	[47]
Photodegradation	kaolinite-supported ZnO	2 g/L	13.0 mg/L	25 °C (± 2 °C)	7.0	60 min	53%	[48]
Photodegradation	Graphene oxide based TiO_2	0.2 g/L	25.0 mg/L	32–42 °C	6.0	240 min	80%	[49]
Biodegradation	HRP@PCB	$[HRP]_0$ = 1 μg/mL	163.0 mg/L	30 °C	7.0	60 min	35%	[43]
Biodegradation	Laccase	2.2 mg/L (200 U/g)	16.3 mg/L	70 °C	6.0	20 min	19%	This work
						60 min	38%	
Catalytic degradation	Tris-Cu	0.44 mg/L				20 min	93%	
						60 min	100%	

M/Z_x: The core–shell bimetallic MIL-101/ZIF-67_x; PMS: Peroxymonosulfate; HRP@PCB: Nanocapsulation of horseradish peroxidase.

Table S5. Comparison of degradation efficiency of phenol by different methods

Degradation method	Catalyst	Catalyst concentration	Substrate concentration	Temperature	pH	Time	Degradation efficiency	Ref.
Biodegradation	SDS-Al_{30}	110 mg/L SDS 100 mg/L Al(III)	—	30 °C	8.5	8 days	92%	[50]
Biodegradation	HRP@PCB	$[HRP]_0$ = 1 μg/mL	163.0 mg/L	30 °C	7.0	60 min	46%	[43]
Biodegradation	Aspergillus awamori cells	1×10^5 conidia / mL medium	1.0 g/L	30 °C	5.5	7–8 days	100%	[46]
Catalytic degradation	DSS400 + PS<br>DSS700 + PS	0.5 g/L	200.0 mg/L	25 °C	7.0	350 min	36%<br>58%	[51]
Photodegradation	TiO_2<br>Boron-doped TiO_2	1 g/L	3.3 mg/L	25 °C	7.0	120 min	48%<br>76%	[52]
Photodegradation	Ag/TiO_2 nanofibers	2.06 g/L<br>0.65 g/L<br>1.59 g/L	0.9 mg/L<br>0.8 mg/L<br>0.8 mg/L	—<br>—<br>—	7.87<br>8.0<br>7.0	10 h	91%<br>85%<br>78%	[53]
Biodegradation	Laccase	2.2 mg/L (200 U/g)	16.3 mg/L	70 °C	6.0	20 min<br>60 min	40%<br>48%	This work
Catalytic degradation	Tris-Cu	0.44 mg/L				20 min<br>60 min	90%<br>100%	

SDS-Al_{30}: Keggin-aluminum nanocluster; HRP@PCB: Nanocapsulation of horseradish peroxidase; DSS400 and DSS700: The mineral-rich biochar produced from sewage sludge at 400 °C and 700 °C, respectively; PS: the persulfate activator.

Table S6. Comparison of degradation efficiency of resorcinol by different methods

Degradation method	Catalyst	Catalyst concentration	Substrate concentration	Temperature	pH	Time	Degradation efficiency	Ref.
Photodegradation	TiO_2/CMK-3	0.15 g/L	100.0 mg/L	25 °C	6.0	150 min	78%	[54]
Photodegradation	TiO_2<br>Boron-doped TiO_2	1 g/L	3.3 mg/L	25 °C	7.0	120 min	88%<br>98%	[52]
Electrocatalytic degradation	CuO	—	475.0 mg/L	25 °C	—	168 h	81%	[55]
Biodegradation	Laccase	2.2 mg/L (200 U/g)	16.3 mg/L	70 °C	6.0	20 min<br>60 min	28%<br>35%	This work
Catalytic degradation	Tris-Cu	0.44 mg/L				20 min<br>60 min	87%<br>100%	

TiO_2/CMK-3: TiO_2 nanoparticles on ordered mesoporous carbon.

Table S7. Comparison of degradation efficiency of 2,6-dimethoxyphenol by different methods

Degradation method	Catalyst	Catalyst concentration	Substrate concentration	Temperature	pH	Time	Degradation efficiency	Ref.
Catalytic degradation	Imidazole-Cu nanozyme	0.1 g/L	100.0 mg/L	25 °C	6.8	200 min	98%	[20]
Biodegradation	P. ostreatus	7.91 g/L	815.0 mg/L	40 °C	2.0	100 h	90%	[56]
Biodegradation	Aspergillus awamori cells	1 × 105 conidia / mL medium	1.0 g/L	30 °C	5.5	7 days	100%	[46]
Biodegradation	Laccase	2.2 mg/L (200 U/g)	16.3 mg/L	70 °C	6.0	30 min	45%	This work
						60 min	55%	
Catalytic degradation	Tris-Cu	0.44 mg/L				30 min	85%	
						60 min	87%	

Table S8. Comparison of degradation efficiency of bisphenol A by different methods

Degradation method	Catalyst	Catalyst concentration	Substrate concentration	Temperature	pH	Time	Degradation efficiency	Ref.
Biodegradation	HRP@PCB	$[HRP]_0$ = 1 μg/mL	163.0 mg/L	30 °C	7.0	60 min	40%	[43]
Photodegradation	GO@B-TiO_2 nanocomposite	1 g/L	10.0 mg/L	25 ± 0.1 °C	5.0	240 min	48%	[57]
Sonocatalytic degradation	CuS/$BaWO_4$ composite	1 g/L	40.0 mg/L	25 °C	—	60 min	95%	[58]
Biodegradation	River water inoculum	—	10.0 mg/L	25 °C	—	28 days	100%	[59]
Photodegradation	Fe^{2+}	0.05 mmol/L			—	20 min	100%	
Biodegradation	Laccase	2.2 mg/L (200 U/g)	16.3 mg/L	70 °C	6.0	20 min	49%	This work
						60 min	55%	
Catalytic degradation	Tris-Cu	0.44 mg/L				20 min	74%	
						60 min	81%	

HRP@PCB: Nanocapsulation of horseradish peroxidase; GO@B-TiO_2 nanocomposite: Boron-doped TiO_2 decorated on graphene oxide photocatalysts.

Table S9. Comparison of degradation rate constant of 2,4-dichlorophenol by different methods

Degradation method	Catalyst	Substrate concentration (mg/L)	k_0 (min^{-1})	Ref.
Catalytic degradation	Fe-TiO_2@Fe_3O_4	20.0	0.0236	[44]
Catalytic degradation	Cu^0-Cu_2O@CNTs composite	50.0	0.187	[60]
Electrocatalytic degradation	Glow discharge electrolysis	100.0	0.0162	[61]
Electrocatalytic degradation	Ti/MMO-BDD/Nb	2.0	0.0032	[62]
Photocatalytic degradation	UV/ferrate (VI) oxidation	20.0	0.0024	[63]
Photocatalytic degradation	TiO_2 nanotube-coated disc flow reactor	5.0	0.0127	[64]
Biodegradation	*Pseudomonas* sp.	16.3	0.0024	[65]
Biodegradation	*Staphylococcus xylosus*	16.3	0.0095	[66]
Catalytic degradation	Tris-Cu	16.3	0.2304	This work

k_0: Degradation rate constant; CNTs: Carbon nanotubes; Ti/MMO: Titanium/mixed metal oxides; BDD/Nb: Boron-doped diamond/Niobium electrode.
